# A Risk Classification System With Five-Gene for Survival Prediction of Glioblastoma Patients

**DOI:** 10.3389/fneur.2019.00745

**Published:** 2019-07-16

**Authors:** Yulin Wang, Xin Liu, Gefei Guan, Weijiang Zhao, Minghua Zhuang

**Affiliations:** ^1^Department of Neurosurgery, The First Affiliated Hospital of Shantou University Medical College, Shantou, China; ^2^Department of Stomatology, The First Affiliated Hospital of Shantou University Medical College, Shantou, China; ^3^Department of Neurosurgery, The First Hospital of China Medical University, Shenyang, China; ^4^Center for Neuroscience, Shantou University Medical College, Shantou, China

**Keywords:** glioblastoma (GBM), WGCNA, risk signature, nomogram, prognosis

## Abstract

**Objective:** Glioblastoma (GBM) is the most common and fatal primary brain tumor in adults. It is necessary to identify novel and effective biomarkers or risk signatures for GBM patients.

**Methods:** Differentially expressed genes (DEGs) between GBM and low-grade glioma (LGG) in TCGA samples were screened out and weight correlation network analysis (WGCNA) was performed to confirm WHO grade-related genes. Five genes were selected via multivariate Cox proportional hazards regression analysis and were used to construct a risk signature. A nomogram composed of the risk signature and clinical characters (age, radiotherapy, and chemotherapy experience) was established to predict 1, 3, 5-year survival rate for GBM patients.

**Results:** One hundred ninety-four DEGs in blue gene module were found to be positively related to WHO grade via WGCNA. Five genes (DES, RANBP17, CLEC5A, HOXC11, POSTN) were selected to construct a risk signature for GBM via R language. This risk signature was identified to independently predict the outcome of GBM patients, as well as stratified by IDH1 status, MGMT promoter status, and radio-chemotherapy. The nomogram was established which combined the risk signature with clinical factors. The results of c-index, ROC curve and calibration plot revealed the nomogram showing a good accuracy for predicting 1, 3, or 5-year survival of GBM patients.

**Conclusion:** The risk signature with five genes could serve as an independent factor for predicting the prognosis of patients with GBM. Moreover, the nomogram with the risk signature and clinical traits proved to perform better for predicting 1, 3, 5-year survival rate.

## Introduction

Glioblastoma (GBM) is the most common and aggressive type of primary brain tumor in adult. Despite comprehensive regimens including maximum surgical resection, radiation therapy and chemotherapy, the prognosis of GBM is notoriously poor, with a median survival of 14 months and the 5-year survival rate remaining at ~5% (1). While intervention of these multimodal treatments cannot eradicate this devastating disease, therapeutic resistance and GBM recurrence were inevitable. Although temozolomide (TMZ) has been proven to prolong the survival of GBM patients as a first-line chemotherapeutic agent, recent studies show that an amount of patients with GBM develop resistance to TMZ during treatment (2), and the recurrence rate of GBM was up to 90% (3). These awful therapeutic outcomes were mainly attributed to glioma stem cells (GSCs) and heterogeneity in GBM (4, 5). Likewise, several new drugs, such as monoclonal antibody targeting epidermal growth factor receptor variant III (EGFRvIII), have been proven to show therapeutic efficiency in some cancers, but not in glioma (6). Since only 30% of GBM cases contain EGFRvIII, this means a majority of GBM patients fail to benefit from EGFRvIII-targeted therapy (7, 8). Therefore, it becomes particularly important to search for novel molecular biomarkers that precisely predict the prognosis and to choose appropriate individualized treatment strategies for patients with GBM.

With the progress of genetics and molecular biology, an increasing number of molecular biomarkers were discovered in glioma, for instance, IDH mutation, MGMT methylation, TERT promoter mutation, EGFR and P53 (9). As is known to all, IDH1/2 mutation and MGMT promoter methylation are two important biomarkers in glioma. IDH mutation mainly exists in low grade glioma and secondary GBM, and associates with prognosis and GBM subtype (10). Moreover, IDH phenotype was also reported to be potent to form a glioma CpG island methylator phenotype (G-CIMP) and to be related to genomic methylation and gene mutation, such as P53 and TERT mutation (10). MGMT promoter methylation accounts for ~40% of GBM samples and associates with favorable prognosis of patients receiving radiotherapy and chemotherapy (11). Interestingly, it has been observed that IDH-mutated gliomas frequently carry MGMT promoter methylation and are sensitive to temozolomide (12). These findings indicate that there are cross talks among these key molecular biomarkers and a single gene cannot completely represent the characters of the glioma, as well as GBM. This may partially explain that GBM patients fail to take more advantages from some targeted small molecule inhibitors application (13). Therefore, risk signatures with correlative biomarkers have been developed, which have shown better performance in GBM treatment and survival prediction (14, 15). In this study, we developed a risk signature with five genes associated with survival of GBM patients. On this basis, a nomogram including the risk signature and clinical factors was established and it proved to be effective in predicting the clinical outcome of patients with GBM.

## Materials and Methods

### Data of Glioma Patients in the Study

Gene expression and survival data of glioma in TCGA were downloaded from GlioVis (http://gliovis.bioinfo.cnio.es/) (16). six hundred twenty samples from TCGA GBMLGG (RNA-seq) were selected for screening differentially expressed genes between GBM and low-grade glioma (LGG). Five hundred twenty-five samples from TCGA GBM (HG-UG133A) were used to construct a clinical survival prediction model and internal validation.

### Identification of Differentially Expressed Genes Between GBM and LGG

Based on 470 lower grade glioma (LGG, World Health Organization [WHO] grade II and III) (17, 18) and 150 GBM samples in TCGA GBMLGG dataset, R language (edgeR package, R version 3.51) was performed to identify differentially expressed genes (DEGs). Genes with |log_2_(fold-change)|> 1 and false discovery rate (FDR) < 0.05 were considered as DEGs for further analysis.

### Weighted Correlation Network Analysis for Discovering Grade-Related Gene Modules

To select glioma grade-related genes from DEGs, we performed weight correlation network analysis (WGCNA) (19). The expression data of DEGs and clinical data (WHO grade, age, gender, IDH status, survival time, and status) were imported and analyzed by R package WGCNA. The genes were classified into several gene modules using an appropriate soft-thresholding power which was calculated by the pickSoftThreshold function (20). The minimum gene size in each module was set as 10. The module eigengenes were calculated and similar modules were clustered and merged according to the module dissection threshold. The correlations between gene modules and clinical traits were calculated and visualized through a heatmap. In this research, we chose the module which is positively related to WHO grade for further study.

### Construction and Evaluation of Risk Signature With Selected Genes

Univariate Cox proportional hazards regression analysis was applied to assess the relationship between the expression of DEGs and the overall survival (OS) of patients with GBM in TCGA GBMLGG (RNA-seq) and HG-UG133A platform, respectively. Common genes with *P* < 0.05 were sorted out and presented as a Venn diagram by R. We then performed multivariate Cox proportional hazards models and filtered the common genes by step function in R. A risk score formula was designed according to the multivariate Cox regression analysis results (18), as follows:

$$\begin{array}{l} \text{Risk score}=\left({\text{expr}}_{\text{gene}1}\times {\text{Coef}}_{\text{gene}1}\right)+\left({\text{expr}}_{\text{gene}2}\times {\text{Coef}}_{\text{gene}2}\right)\\ \;+\ldots +\left({\text{expr}}_{\text{genen}}\times {\text{Coef}}_{\text{genen}}\right) \end{array}$$

The patients were divided into low-risk and high-risk groups according to the median risk score value. KM survival analysis and time-dependent receiver operating characteristic (ROC) curve analysis were used to evaluate the prognostic value.

### Bioinformatics Analysis

DEGs between low-risk and high-risk groups with FDR <0.05 were filtered by R language (edgeR package) and used for Gene ontology (GO) and KEGG pathway analysis via DAVID website (https://david.ncifcrf.gov/) (21). GO terms (FDR < 0.05) and KEGG pathways (*P*-value <0.05) were screened out and visualized via R package ggplot2. Gene set enrichment analysis (GSEA, http://software.broadinstitute.org/gsea/index.jsp) were used to confirm the GO terms and KEGG pathways in the low-risk and high-risk groups (22). Normalized enrichment score (NES) and FDR were calculated to verify the statistical difference for GSEA analysis.

### Construction and Evaluation of Clinical Survival Prediction Model

By combining with clinical data, a nomogram of clinical survival prediction model was established by using the package of “rms” in R. Samples from TCGA HG-UG133A platform were divided into training cohort (accounting for 70%) and validation cohort (accounting for 30%) by randomly using R package “caret.” The inclusion criteria for data extraction in the predictive model were patients diagnosed with WHO grade IV glioma (GBM). The exclusion criteria included patients with incomplete data such as survival status and time, radiotherapy, and chemotherapy records. The training cohort was used to construct the nomogram of clinical survival prediction model, and the validation cohort was applied for internal validation. Concordance index (C-index), ROC curve analysis and calibration curve were used to measure the performance of the nomogram, which were conducted by R.

### Statistical Analysis

Risk scores of the samples in GBM subtype, IDH1 status, and MGMT promoter were presented as mean ± standard deviation and calculated by Graphpad Prism 8.0. Statistical differences between and among groups were examined by two tailed *t*-test and one-way analysis of variance (ANOVA) followed by Dunnett's post-test, respectively. Kaplan-Meier survival analysis and Cox proportion hazards regression model were conducted with R and R package. *P* < 0.05 was regarded as statistically significant.

## Results

### Identification of Differentially Expressed Genes Between GBM and LGG

**Figure 1:**
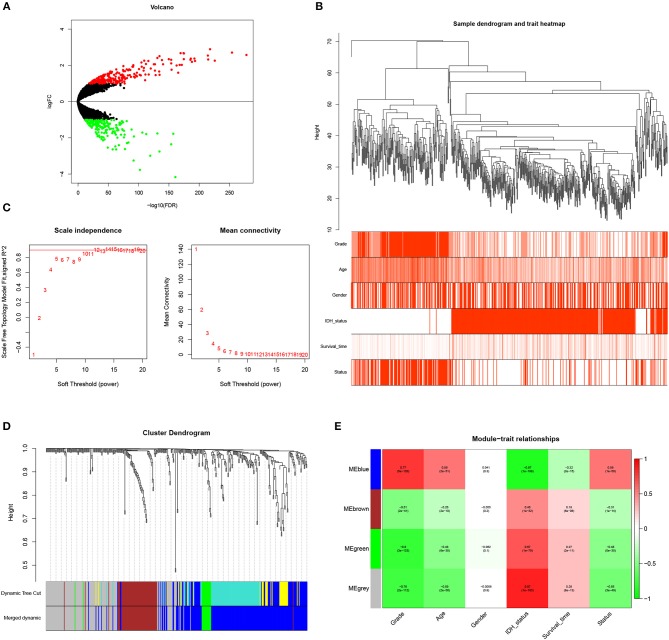
Identification of WHO grade-related genes in glioma. **(A)** Volcano plot showed the distribution of DEGs. **(B)** Sample clusters showed basic clinical information of glioma patients. **(C)** The soft threshold power was calculated and 10 was selected as the power value. **(D)** Similar modules were merged and four modules were generated. **(E)** Heatmap exhibited the relationships between gene modules and clinical traits by Pearson correlation.

GBM is one of the devastating malignancies with poor prognosis. To better construct a survival prediction signature for patients with GBM, we searched for differentially expressed genes between GBM and LGG in TCGA GBMLGG (RNA-seq) dataset. Genes with |log_2_FC|> 1 and FDR < 0.05 were chosen as DEGs. Four hundred eight genes (including 211 up-regulated genes and 197 down-regulated genes) were identified (Figure 1A; Supplementary Table 1).

### WGCNA Analysis Revealed Blue Gene Module Was Related to Glioma Grade

To identify genes associated with clinical traits, we collected the RNA-seq data of DEGs and clinical information (WHO grade, age, gender, IDH status, survival time and status), and performed WGCNA analysis. Firstly, the samples were clustered and basic clinical traits were displayed (Figure 1B). A soft threshold power was then calculated and 10 was selected as the power value to produce a hierarchical clustering tree (Figure 1C). The module dissection threshold was set at 0.15 to merge similar modules and 4 modules were generated (Figure 1D). The relationships between gene modules and clinical traits were confirmed by Pearson correlation and exhibited in a heatmap (Figure 1E). Among the modules, blue gene module contained 194 genes and was the most positively related to WHO grade (*r* = 0.77, *P* <0.0001). In addition, the blue gene module was also correlated with age (*r* = 0.56), IDH status (*r* = −0.87), survival time (*r* = −0.32), and survival status (*r* = 0.56). Therefore, genes in the blue module were used for further study.

### Construction of the Risk Signature With Five-Gene in GBM Cohorts

**Figure 2:**
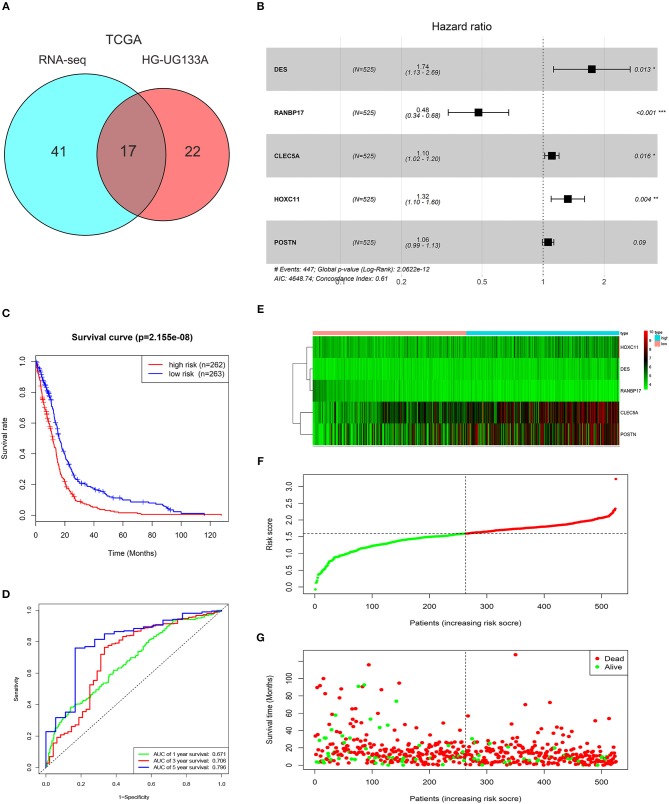
Characteristics of the risk signature with five genes. **(A)** Venn diagram showed 17 common genes correlated with overall survival (*P* < 0.05) between TCGA GBMLGG (RNA-seq) and HG-UG133A platforms. **(B)** Multivariable Cox regression analysis was performed and five genes (DES, RANBP17, CLEC5A, HOXC11, POSTN) were selected to construct the risk signature. **(C)** Difference of overall survival between low-risk and high-risk groups (*P* < 0.0001). **(D)** ROC analysis of 1, 3, 5-year survival according to the five-gene risk signature. **(E)** The expression levels of the five genes (DES, RANBP17, CLEC5A, HOXC11, POSTN) in the signature. **(F)** The distribution of the five-gene signature risk score for each patient. **(G)** The survival time of each patient with GBM and their survival status.

To select prognosis related genes, we performed univariate Cox proportional hazards regression analysis to analyze the genes in blue module in TCGA GBMLGG (RNA-seq) and HG-UG133A platforms. Seventeen overlapped genes significantly correlated with overall survival (*P* < 0.05) between the two platforms were obtained (Figure 2A; Supplementary Table 2). Next, multivariable Cox regression analysis was implemented to filter and optimize the genes for constructing risk signature. Five genes (DES, RANBP17, CLEC5A, HOXC11, POSTN) were screened out, among which RANBP17 was defined as protective with HR <1, whereas others were defined as risky with HR > 1 (Figure 2B). The risk-score formula was constructed as follows: risk score = (0.5536 × expression level of DES) + (−0.7340 × expression level of RANBP17) + (0.0995 × expression level of CLEC5A) + (0.2810 × expression level of HOXC11) + (0.0566 × expression level of POSTN). The risk score for each patient in TCGA HG-UG133A platform was calculated (mean ±SD, 1.5290 ± 0.4039; Quartiles were 1.3257 at 25%, 1.5936 at 50%, and 1.7968 at 75%, respectively) and all the 525 patients were divided into high-risk or low-risk groups based on the median cutoff value of the scores. As shown in Figure 2C, GBM patients with high risk scores indicated poor prognosis. The AUC for the five-gene signature risk score model at 1, 3, and 5-year survival were 0.671, 0.706, and 0.796, respectively (Figure 2D). The results indicated that the risk signature can better predict 1, 3, and 5-year survival for GBM patients. With the increase of risk score, the expression level of RANBP17 was down-regulated, and the expression level of the other 4 genes were up-regulated (Figures 2E,F). In the mean-time, the number of alive patients decreased (Figure 2G).

### Application of the Risk Signature in Stratified GBM Cohorts

**Figure 3:**
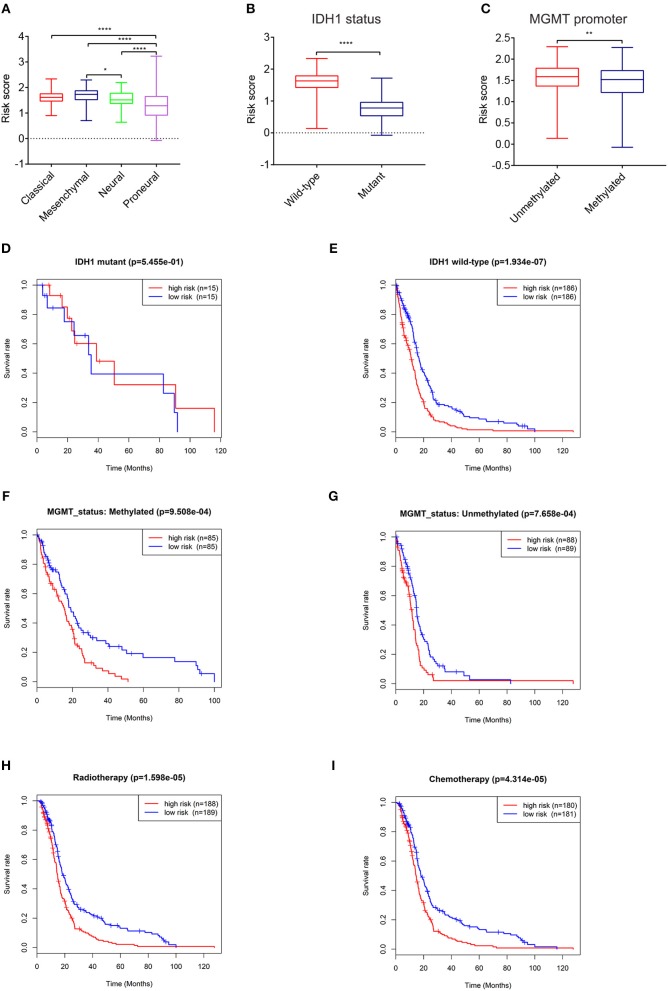
Expression and prognostic significance of the risk signature in different cohorts. Association between the risk signature and different cohorts stratified by molecular subtype **(A)**, IDH1 **(B)**, and MGMT status **(C)** (^*^P < 0.05, ^**^P < 0.01, ^****^P < 0.0001). Prognostic significance of the risk signature in different cohorts stratified by IDH1 status **(D,E)**, MGMT status **(F,G)**, radiotherapy **(H)**, and chemotherapy **(I)**.

To further explore its clinical application, we investigated the relationship between the risk score and glioma subtype, IDH1 and MGMT promoter status, respectively. The mesenchymal subtype inclined to have higher risk scores than neural and proneural subtype (Figure 3A). The risk scores of patients with IDH1 mutant type were lower than IDH1 wild type (Figure 3B). This result was in accordance with the conclusion that IDH1 mutant in glioma was related to better patient prognosis (23). For MGMT promoter, the risk scores decreased in patients with methylated status (*P* < 0.01, Figure 3C), though the average risk scores between the two groups didn't differ largely.

The relationships between the risk score and patient prognosis stratified by IDH1, MGMT promoter status were also explored. There was no significant statistical difference between high-risk group and low-risk group in GBM patients with IDH1 mutant (Figure 3D). This result might be mainly due to the insufficient number of patients. In IDH1 wild-type cohort, patients with low risk scores exhibited longer survival time than high risk group (Figure 3E). In terms of MGMT promoter, no matter of methylated or unmethylated state, the high-risk group indicated dismal prognosis compared with the low-risk group (Figures 3F,G). Furthermore, in consideration of the importance of radio- and chemo-therapy in the treatment of glioma, we analyzed the association between the risk score and the response to standard radio- and chemo-therapy. The patients with low risk scores exhibited favorable prognosis in either radiotherapy or chemotherapy (Figures 3H,I). These results revealed that the risk signature could serve as an independent factor for predicting the prognosis of patients with GBM.

### Functional Analysis of the Five Genes in the Risk Signature

**Figure 4:**
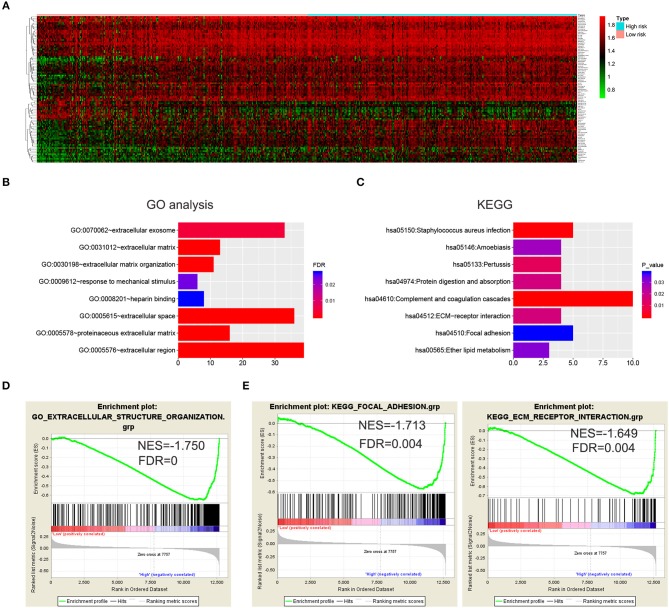
Biological functions and KEGG pathways related to the risk signature with five genes. **(A)** Heatmap showed the DEGs between the high-risk group and the low-risk group (FDR < 0.05). GO analysis **(B)** and KEGG pathway analysis **(C)** via DAVID based on the DEGs. **(D,E)** GSEA was performed to confirm the GO term (extracellular structure organization) and KEGG pathways (ECM receptor interaction and focal adhesion).

To further investigate the functional roles and KEGG pathways associated with the risk signature, we first screened out the DEGs between the high-risk group and low-risk group. Ninety-five genes with FDR <0.05 were selected for GO and KEGG pathway analysis via DAVID (Figure 4A; Supplementary Table 3). We discovered that the five-gene risk signature was functionally associated with extracellular matrix related terms, including extracellular exosome, extracellular matrix, and extracellular matrix organization (FDR < 0.05, Figure 4B). Correspondingly, several KEGG pathways (*P* < 0.05) such as ECM-receptor interaction and focal adhesion pathways were also obtained (Figure 4C). To further confirm these results, the samples were divided into high-risk and low-risk groups according to the median of risk scores, and GSEA were applied. Similar GO terms and KEGG pathways were observed via GSEA analysis (Figures 4D,E). Collectively, these results revealed that the five-gene risk signature was correlated to extracellular matrix and cell adhesion functions, which play vital roles in glioma invasion and progression (24, 25).

### Construction of a Clinical Survival Prediction Model via the Risk Signature Combined With Clinicopathologic Features

**Figure 5:**
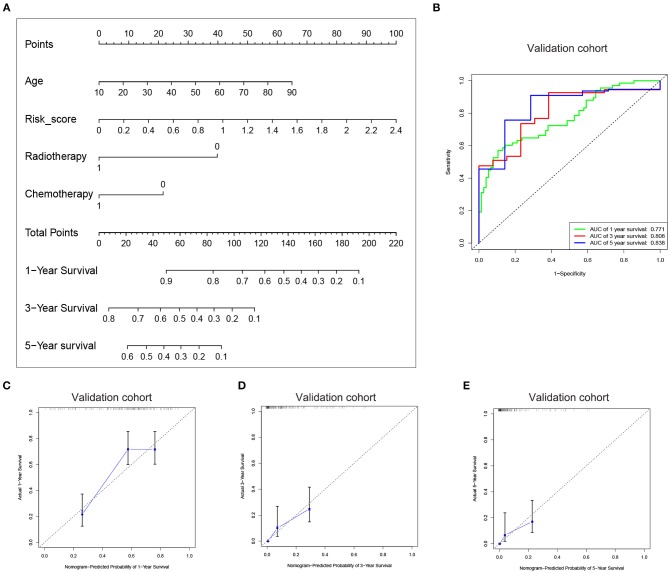
Construction of the nomogram based on the risk signature with five-gene. **(A)** The nomogram was constructed for predicting 1, 3, 5-year survival rate of GBM patients. **(B)** ROC curve was used to evaluate the efficiency of the clinical predictive model. **(C–E)** The calibration curves for predicting patient survival at 1, 3, and 5 years in the validation cohort.

Since the risk signature had a better performance in predicting the prognosis of GBM patients, we explored its clinical significance combining with clinical characters (age, radiotherapy, and chemotherapy experience). Firstly, the samples in the TCGA HG-UG133A platform were divided into training cohort (364 cases) and validation cohort (155 cases) randomly (Table 1). Then, multivariable Cox regression analysis was performed to assess the selected variable's contribution in predicting prognosis of GBM patients. The results indicated that the factors, such as risk score, age, acceptance of radiotherapy and chemotherapy, were correlated with patients' survival significantly both in training cohort and validation cohort (Table 2). A clinical survival prediction model was constructed based on the data in training cohort and presented in a nomogram for predicting 1, 3, 5-year survival (Figure 5A). C-index, ROC curve and calibration plot were used to evaluate the efficiency of the clinical predictive model. The C-indexes in training cohort and validation cohort were 0.729 and 0.708, respectively. The area under the curves (AUC) of the nomogram for 1, 3, 5-year-survival were 0.771, 0.808, and 0.838 in validation cohort, respectively (Figure 5B). In the training set, the area under the curves (AUC) for 1, 3, 5-year-survival were 0.796, 0.79, and 0.851, respectively (Supplementary Figure 1A). The calibration plot for the probability of survival at 1, 3, or 5-years showed an optimal agreement between the prediction and observation, both in the validation cohort (Figures 5C–E) and training cohort (Supplementary Figures 1B–D). These results above revealed that the nomogram demonstrated a good accuracy for predicting 1, 3, or 5-year survival of GBM patients.

**Table 1:** Demographics and clinicopathologic characteristics of patients in training cohort and validation cohort.

**Characteristic**	**Training cohort (*****n*** **=** **364)**		**Validation cohort (*****n*** **=** **155)**	
	**No. of patients**	**%**	**No. of patients**	**%**
**RISK SCORE**				
Median	1.5332		1.5356	
Range	0.1301 to 2.2929		−0.0730 to 3.2246	
**AGE, YEARS**				
Median	59		57	
Range	15–89		11–89	
**RADIOTHERAPY**				
Yes	260	71.4286	118	76.1290
No	104	28.5714	37	23.8710
**CHEMOTHERAPY**				
Yes	246	67.5824	115	74.1935
No	118	32.4176	40	25.8065

**Table 2:** Multivariate analysis of the training cohort and validation cohort for overall survival.

**Variable**	**Training cohort**			**Validation cohort**		
	***P***	**HR**	**95% CI**	***P***	**HR**	**95% CI**
Risk score	<0.0001	2.4617	1.7110–3.5417	0.0043	1.9083	1.2246–2.9738
Age (years)	0.0004	1.0177	1.0079–1.0276	0.001	1.0244	1.0098–1.0392
Radiotherapy yes vs. no	<0.0001	0.4230	0.3139–0.5701	0.0013	0.4827	0.3099–0.7518
Chemotherapy yes vs. no	0.0011	0.6274	0.4740–0.8304	0.0159	0.5825	0.3755–0.9036

## Discussion

So far, GBM is still a lethal disease without efficient therapeutic regimens. The failure to develop new treatments ascribes to a lack of validation of novel molecular targets, which are often performed in animal models and directly translated to human trials (26). Thus, exploration and validation novel molecular targets are not only necessary, but also very urgent. In the present study, we identified DEGs between GBM and LGG in TCGA data, and confirmed 17 genes significantly correlated with prognosis. Finally, five genes (DES, RANBP17, CLEC5A, HOXC11, POSTN) were selected to construct a risk signature for GBM. Among the five genes, POSTN is an ECM protein and is involved in various cellular processes, including epithelial-mesenchymal transition (EMT) and cell migration (27). POSTN is highly expressed in glioma tissues and has been considered as a biomarker of glioma malignancy and recurrence (28, 29). It has also been reported that POSTN recruits M2 tumor-associated macrophages and promotes glioma stem cells (GSCs) growth (30). CLEC5A is a spleen tyrosine kinase-coupled receptor, which is abundantly expressed in monocytes, macrophages and neutrophils, and critical for inflammation response (31, 32). A recent study has shown that CLEC5A is upregulated in GBM significantly and is associated with poor prognosis (33). Furthermore, downregulation of CLEC5A can inhibit the capabilities of proliferation, migration, and invasion, and promotes apoptosis and G1 arrest in GBM cell lines (33). These results are consistent with our findings that the expression level of CLEC5A increased with the ascent of risk scores and CLEC5A was a risk factor in GBM. Although few have been reported about the other three genes in glioma, they have vital functions and might serve as potential targets for GBM. For instance, DES encodes the intermediate filament protein desmin, which is expressed in cardiac, skeletal, and smooth muscle cells, and its mutations can cause isolated cardiomyopathies and cardiac conduction diseases (34, 35). A recent study demonstrated that desmin loss is observed in 92% malignant mesothelioma samples, 76% malignant effusions, 29% benign mesothelial hyperplasia tissues, but not in the reactive effusions (36). Thus, desmin may serve as a useful biomarker in the discrimination between reactive mesothelial proliferation and malignant mesothelioma. As a RanGTP-binding protein, RANBP17 belongs to the importin beta family and is preferentially expressed in the testis (37, 38). RANBP17 is upregulated in dilated cardiomyopathy and ischemic cardiomyopathy samples, and may regulate the transport of different cargos in specific cardiomyopathies through enhancing the transcriptional activation of the EA2 transcription factors E12 and E47 (39). HOXC11 belongs to homeobox superfamily that are responsible for encoding transcription factors regulating development (40). HOXC6 and HOXC11 have been shown to induce differentiation of GOTO neuroblastoma cells into Schwannian cells via transcription activation of S100β (41). Moreover, HOXC11 is found to be closely correlated with the survival of patients with renal cell cancer, cervical cancer or breast cancer, and serves as a therapeutic target (40, 42). This risk signature comprised of the five genes was identified to significantly correlate with the survival of GBM patients, as well as stratified by IDH1 status, MGMT promoter status, and radiochemotherapy. In addition, GO and KEGG pathway analysis were applied via DAVID and GSEA, and elucidated that the five-gene risk signature was mainly related to extracellular matrix and cell adhesion function. EMC organization and cell adhesion are indispensable biological processes in tumor development and progression (43, 44), indicating the essential value of our signature.

Nomograms have been applied extensively and exhibit favorable effects on predicting clinical risk signatures and outcomes in some cancers (45, 46). For better clinical application, we combined the risk signature with clinical factors (age, radiotherapy, and chemotherapy experience) and established a nomogram, which was validated to have better performance for predicting the outcomes of patients with GBM. The nomogram contained four items, and predicted the 1, 3, 5-year survival rate based on the sum of the score in each item. This clinical prediction model aimed at precisely predicting the prognosis of GBM patients and corresponded to the idea of individual treatment. However, there were some deficiencies in this study. Firstly, the sample size was limited. Five hundred nineteen samples were incorporated, and 364 cases were used for constructing model. The second limitation was that the samples were downloaded from TCGA, and it didn't contain information about extent of tumor resection, which is a key factor closely related to survival time in patients with GBM (47). A collection of detailed clinical records and further validation should be carried out in future study. Despite the above shortcomings, this study still has its advantages and innovations. Firstly, we performed an accurate and widely used method, WGCNA (48), and confirmed genes associated with glioma grade, which is an clinical indicator directly associated with the prognosis of glioma patients. Secondly, the risk signature with five genes was proven to be an independent prognostic biomarker in GBM via Kaplan-Meier survival analysis and multivariable Cox regression analysis. In addition, on the basis of the risk signature and other clinical factors (age, radiotherapy, and chemotherapy experience), the nomogram can predict the 1, 3, 5-year survival rate precisely, thus providing evidences of treatment for GBM patients. Altogether, our study indicated the potential value of our model for predicting the survival of GBM patients.

## Data Availability

Publicly available datasets were analyzed in this study. This data can be found here: http://gliovis.bioinfo.cnio.es/.

## Author Contributions

YW: conceptualization and writing-original draft. YW and GG: methodology. YW and XL: project administration. MZ: supervision. WZ and MZ: writing-review and editing.

### Conflict of Interest Statement

The authors declare that the research was conducted in the absence of any commercial or financial relationships that could be construed as a potential conflict of interest.

## References

[1] Delgado-Lopez PD Corrales-Garcia EM. Survival in glioblastoma: a review on the impact of treatment modalities. Clin Transl Oncol. (2016) 18:1062–71. 10.1007/s12094-016-1497-x 26960561

[2] Dai S Yan Y Xu Z Zeng S Qian L Huo L. SCD1 confers temozolomide resistance to human glioma cells via the Akt/GSK3beta/beta-catenin signaling axis. Front Pharmacol. (2017) 8:960. 10.3389/fphar.2017.00960 29354058 PMC5758607

[3] Hager J Herrmann E Kammerer S Dinc N Won SY Senft C. Quick-weller: impact of resection on overall survival of recurrent glioblastoma in elderly patients. Clin Neurol Neurosurg. (2018) 174:21–5. 10.1016/j.clineuro.2018.08.033 30195896

[4] Hombach-Klonisch S Mehrpour M Shojaei S Harlos C Pitz M Hamai A. Glioblastoma and chemoresistance to alkylating agents: involvement of apoptosis, autophagy, and unfolded protein response. Pharmacol Ther. (2018) 184:13–41. 10.1016/j.pharmthera.2017.10.017 29080702

[5] Auffinger B Spencer D Pytel P Ahmed AU Lesniak MS. The role of glioma stem cells in chemotherapy resistance and glioblastoma multiforme recurrence. Expert Rev Neurother. (2015) 15:741–52. 10.1586/14737175.2015.1051968 26027432 PMC4830899

[6] Raucher D Dragojevic S Ryu J. Macromolecular drug carriers for targeted glioblastoma therapy: preclinical studies, challenges, and future perspectives. Front Oncol. (2018) 8:624. 10.3389/fonc.2018.00624 30619758 PMC6304427

[7] Chistiakov DA Chekhonin IV Chekhonin VP. The EGFR variant III mutant as a target for immunotherapy of glioblastoma multiforme. Eur J Pharmacol. (2017) 810:70–82. 10.1016/j.ejphar.2017.05.064 28583430

[8] Paff M Alexandru-Abrams D Hsu FP Bota DA. The evolution of the EGFRvIII (rindopepimut) immunotherapy for glioblastoma multiforme patients. Hum Vaccin Immunother. (2014) 10:3322–31. 10.4161/21645515.2014.983002 25625931 PMC4514075

[9] Ludwig K Kornblum HI. Molecular markers in glioma. J Neurooncol. (2017) 134:505–12. 10.1007/s11060-017-2379-y 28233083 PMC5568999

[10] Karsy M Guan J Cohen AL Jensen RL Colman H. New molecular considerations for glioma: IDH, ATRX, BRAF, TERT, H3 K27M. Curr Neurol Neurosci Rep. (2017) 17:19. 10.1007/s11910-017-0722-5 28271343

[11] Chen R Smith-Cohn M Cohen AL Colman H. Glioma subclassifications and their clinical significance. Neurotherapeutics. (2017) 14:284–97. 10.1007/s13311-017-0519-x 28281173 PMC5398991

[12] Abe H Natsumeda M Kanemaru Y Watanabe J Tsukamoto Y Okada M. MGMT expression contributes to temozolomide resistance in H3K27M-mutant diffuse midline gliomas and MGMT silencing to temozolomide sensitivity in IDH-mutant gliomas. Neurol Med Chir. (2018) 58:290–5. 10.2176/nmc.ra.2018-0044 29848907 PMC6048353

[13] Brandes AA Carpentier AF Kesari S Sepulveda-Sanchez JM Wheeler HR Chinot O. A Phase II randomized study of galunisertib monotherapy or galunisertib plus lomustine compared with lomustine monotherapy in patients with recurrent glioblastoma. Neuro Oncol. (2016) 18:1146–56. 10.1093/neuonc/now009 26902851 PMC4933481

[14] Gao WZ Guo LM Xu TQ Yin YH Jia F. Identification of a multidimensional transcriptome signature for survival prediction of postoperative glioblastoma multiforme patients. J Transl Med. (2018) 16:368. 10.1186/s12967-018-1744-8 30572911 PMC6302404

[15] Capper D von Deimling A Brandes AA Carpentier AF Kesari S Sepulveda-Sanchez JM. Biomarker and histopathology evaluation of patients with recurrent glioblastoma treated with galunisertib, lomustine, or the combination of galunisertib and lomustine. Int J Mol Sci. (2017) 18:E995. 10.3390/ijms18050995 28481241 PMC5454908

[16] Bowman RL Wang Q Carro A Verhaak RG Squatrito M. GlioVis data portal for visualization and analysis of brain tumor expression datasets. Neuro Oncol. (2017) 19:139–41. 10.1093/neuonc/now247 28031383 PMC5193031

[17] Dworkin M Mehan W Niemierko A Kamran SC Lamba N Dietrich J. Increase of pseudoprogression and other treatment related effects in low-grade glioma patients treated with proton radiation and temozolomide. J Neurooncol. (2018) 142:69–77. 10.1007/s11060-018-03063-1 30488294

[18] Qian Z Li Y Fan X Zhang C Wang Y Jiang T. Molecular and clinical characterization of IDH associated immune signature in lower-grade gliomas. Oncoimmunology. (2018) 7:e1434466. 10.1080/2162402x.2018.1434466 29872572 PMC5980422

[19] Langfelder P Horvath S. WGCNA: an R package for weighted correlation network analysis. BMC Bioinformatics. (2008) 9:559. 10.1186/1471-2105-9-559 19114008 PMC2631488

[20] Zhang X Feng H Li Z Li D Liu S Huang H. Application of weighted gene co-expression network analysis to identify key modules and hub genes in oral squamous cell carcinoma tumorigenesis. Onco Targets Ther. (2018) 11:6001–21. 10.2147/ott.s171791 30275705 PMC6157991

[21] Huang da W Sherman BT Lempicki RA. Systematic and integrative analysis of large gene lists using DAVID bioinformatics resources. Nat Protoc. (2009) 4:44–57. 10.1038/nprot.2008.211 19131956

[22] Subramanian A Tamayo P Mootha VK Mukherjee S Ebert BL Gillette MA. Gene set enrichment analysis: a knowledge-based approach for interpreting genome-wide expression profiles. Proc Natl Acad Sci USA. (2005) 102:15545–50. 10.1073/pnas.0506580102 16199517 PMC1239896

[23] Seliger C Luber C Gerken M Schaertl J Proescholdt M Riemenschneider MJ. Use of metformin and survival of patients with high-grade glioma. Int J Cancer. (2019) 144:273–80. 10.1002/ijc.31783 30091464

[24] Gritsenko PG Friedl P. Adaptive adhesion systems mediate glioma cell invasion in complex environments. J Cell Sci. (2018) 131:jcs216382. 10.1242/jcs.216382 29991514 PMC6104823

[25] Ferrer VP Moura Neto V Mentlein R. Glioma infiltration and extracellular matrix: key players and modulators. Glia. (2018) 66:1542–65. 10.1002/glia.23309 29464861

[26] Caponegro MD Moffitt RA Tsirka SE. Expression of neuropilin-1 is linked to glioma associated microglia and macrophages and correlates with unfavorable prognosis in high grade gliomas. Oncotarget. (2018) 9:35655–65. 10.18632/oncotarget.26273 30479695 PMC6235016

[27] Park SY Piao Y Jeong KJ Dong J de Groot JF. Periostin (POSTN) regulates tumor resistance to antiangiogenic therapy in glioma models. Mol Cancer Ther. (2016) 15:2187–97. 10.1158/1535-7163.Mct-15-0427 27307601 PMC5104278

[28] Mikheev AM Mikheeva SA Trister AD Tokita MJ Emerson SN Parada CA. Periostin is a novel therapeutic target that predicts and regulates glioma malignancy. Neuro Oncol. (2015) 17:372–82. 10.1093/neuonc/nou161 25140038 PMC4483094

[29] Tian B Zhang Y Zhang J. Periostin is a new potential prognostic biomarker for glioma. Tumour Biol. (2014) 35:5877–83. 10.1007/s13277-014-1778-3 24719188

[30] Zhou W Ke SQ Huang Z Flavahan W Fang X Paul J. Periostin secreted by glioblastoma stem cells recruits M2 tumour-associated macrophages and promotes malignant growth. Nat Cell Biol. (2015) 17:170–82. 10.1038/ncb3090 25580734 PMC4312504

[31] Chen ST Li FJ Hsu TY Liang SM Yeh YC Liao WY. CLEC5A is a critical receptor in innate immunity against Listeria infection. Nat Commun. (2017) 8:299. 10.1038/s41467-017-00356-3 28824166 PMC5563510

[32] Sprokholt J Helgers LC Geijtenbeek TB. Innate immune receptors drive dengue virus immune activation and disease. Future Virol. (2017) 13:287–305. 10.2217/fvl-2017-0146 29937918 PMC6004600

[33] Fan HW Ni Q Fan YN Ma ZX Li YB. C-type lectin domain family 5, member A (CLEC5A, MDL-1) promotes brain glioblastoma tumorigenesis by regulating PI3K/Akt signalling. Cell Prolif . (2019) 52:e12584. 10.1111/cpr.12584 30834619 PMC6536598

[34] Bouvet M Dubois-Deruy E Alayi TD Mulder P El Amranii M Beseme O. Increased level of phosphorylated desmin and its degradation products in heart failure. Biochem Biophys Rep. (2016) 6:54–62. 10.1016/j.bbrep.2016.02.014 28955862 PMC5600436

[35] Brodehl A Gaertner-Rommel A Milting H. Molecular insights into cardiomyopathies associated with desmin (DES) mutations. Biophys Rev. (2018) 10:983–1006. 10.1007/s12551-018-0429-0 29926427 PMC6082305

[36] Onder S Ozogul E Koksal D Sarinc Ulasli S Firat P Emri S. Diagnostic value of BAP1, GLUT-1 and desmin expression in the discrimination between reactive mesothelial proliferation and malignant mesothelioma in tissues and effusions. Cytopathology. (2019). 10.1111/cyt.12738. [Epub ahead of print]. 31165505

[37] Bao J Wu Q Song R Jie Z Zheng H Xu C. RANBP17 is localized to the XY body of spermatocytes and interacts with SPEM1 on the manchette of elongating spermatids. Mol Cell Endocrinol. (2011) 333:134–42. 10.1016/j.mce.2010.12.021 21184802 PMC3039071

[38] Kutay U Hartmann E Treichel N Calado A Carmo-Fonseca M Prehn S. Identification of two novel RanGTP-binding proteins belonging to the importin beta superfamily. J Biol Chem. (2000) 275:40163–8. 10.1074/jbc.M006242200 11024021

[39] Molina-Navarro MM Rosello-Lleti E Tarazon E Ortega A Sanchez-Izquierdo D Lago F. Heart failure entails significant changes in human nucleocytoplasmic transport gene expression. Int J Cardiol. (2013) 168:2837–43. 10.1016/j.ijcard.2013.03.192 23651824

[40] Liu YJ Zhu Y Yuan HX Zhang JP Guo JM Lin ZM. Overexpression of HOXC11 homeobox gene in clear cell renal cell carcinoma induces cellular proliferation and is associated with poor prognosis. Tumour Biol. (2015) 36:2821–9. 10.1007/s13277-014-2909-6 25476856

[41] Zhang X Hamada J Nishimoto A Takahashi Y Murai T Tada M. HOXC6 and HOXC11 increase transcription of S100beta gene in BrdU-induced *in vitro* differentiation of GOTO neuroblastoma cells into Schwannian cells. J Cell Mol Med. (2007) 11:299–306. 10.1111/j.1582-4934.2007.00020.x 17488478 PMC3822828

[42] Sun B Hua J Cui H Liu H Zhang K Zhou H. MicroRNA-1197 downregulation inhibits proliferation and migration in human non- small cell lung cancer cells by upregulating HOXC11. Biomed Pharmacother. (2019) 117:109041. 10.1016/j.biopha.2019.109041 31181445

[43] Benthani FA Herrmann D Tran PN Pangon L Lucas MC Allam AH. ‘MCC' protein interacts with E-cadherin and beta-catenin strengthening cell-cell adhesion of HCT116 colon cancer cells. Oncogene. (2018) 37:663–672. 10.1038/onc.2017.362 29035389

[44] Giussani M Triulzi T Sozzi G Tagliabue E. Tumor extracellular matrix remodeling: new perspectives as a circulating tool in the diagnosis and prognosis of solid tumors. Cells. (2019) 8:E81. 10.3390/cells8020081 30678058 PMC6406979

[45] Dong D Tang L Li ZY Fang MJ Gao JB Shan XH. Development and validation of an individualized nomogram to identify occult peritoneal metastasis in patients with advanced gastric cancer. Ann Oncol. (2019) 30:431–8. 10.1093/annonc/mdz001 30689702 PMC6442651

[46] Caulfield S Menezes G Marignol L Poole C. Nomograms are key decision-making tools in prostate cancer radiation therapy. Urol Oncol. (2018) 36:283–292. 10.1016/j.urolonc.2018.03.017 29680180

[47] Hameed NUF Qiu T Zhuang D Lu J Yu Z Wu S Transcortical insular glioma resection: clinical outcome and predictors. J Neurosurg. (2018) 1:1–11. 10.3171/2018.4.Jns18424 30485243

[48] Pei G Chen L Zhang W. WGCNA application to proteomic and metabolomic data analysis. Methods Enzymol. (2017) 585:135–158. 10.1016/bs.mie.2016.09.016 28109426

