# Supplementary material for: A Risk Classification System With Five-Gene for Survival Prediction of Glioblastoma Patients
Source: Front Neurol. 2019 Jul 16;10:745. doi: 10.3389/fneur.2019.00745 (PMC6646669; doi:10.3389/fneur.2019.00745)
Supplement: Supplementary file 1 [file Table_1.docx]

Supplementary Table1. Differentially expressed genes between LGG and GBM in TCGA RNA-seq dataset.

| Gene | logFC | logCPM | P Value | FDR |
| --- | --- | --- | --- | --- |
| ABCC3 | 1.005709 | 5.885825 | 4.93E-80 | 9.14E-78 |
| ADAM29 | -1.73274 | 4.816648 | 1.89E-45 | 1.10E-43 |
| ADAMDEC1 | 1.341546 | 5.155487 | 4.42E-80 | 8.28E-78 |
| ADAMTS20 | -1.81838 | 5.198305 | 8.84E-74 | 1.32E-71 |
| ADH1B | -1.20369 | 5.278955 | 5.33E-44 | 2.86E-42 |
| AGTR1 | 1.005786 | 4.991671 | 1.57E-37 | 6.21E-36 |
| ALOX12B | -1.57912 | 4.991061 | 2.09E-48 | 1.38E-46 |
| AMY1A | -2.05164 | 4.558503 | 5.60E-41 | 2.63E-39 |
| AMY2A | -1.97354 | 4.653021 | 1.27E-44 | 7.10E-43 |
| ANKK1 | 1.051502 | 4.618608 | 1.74E-25 | 3.78E-24 |
| ANKRD34C | -1.13804 | 4.91552 | 6.95E-27 | 1.59E-25 |
| ANXA2P1 | 1.123653 | 5.331807 | 8.20E-68 | 1.02E-65 |
| APCDD1L | 1.674888 | 4.741264 | 4.64E-78 | 7.99E-76 |
| AQP5 | 1.360392 | 5.245525 | 1.22E-90 | 2.75E-88 |
| AQP7 | -1.86918 | 4.573095 | 4.05E-37 | 1.58E-35 |
| AREG | 1.612846 | 4.69595 | 8.55E-68 | 1.05E-65 |
| ARL9 | 1.462986 | 4.915339 | 1.85E-73 | 2.74E-71 |
| ASAH2B | -1.21407 | 5.030317 | 2.63E-34 | 8.85E-33 |
| ATP1A4 | -1.04493 | 4.839317 | 7.21E-22 | 1.24E-20 |
| ATP6V0D2 | 1.524421 | 4.577944 | 3.31E-51 | 2.45E-49 |
| BAGE2 | -3.24659 | 4.778204 | 8.46E-95 | 2.07E-92 |
| BARX1 | 1.595866 | 4.592201 | 2.50E-57 | 2.28E-55 |
| BEND4 | -1.04127 | 5.513733 | 1.16E-43 | 6.14E-42 |
| BTBD8 | -1.4982 | 5.211113 | 3.34E-57 | 3.03E-55 |
| C10orf81 | 1.025068 | 5.009124 | 3.12E-39 | 1.35E-37 |
| C11orf88 | 1.414414 | 4.555206 | 2.65E-42 | 1.34E-40 |
| C15orf26 | 1.000505 | 4.700563 | 8.03E-26 | 1.76E-24 |
| C15orf28 | -1.01055 | 4.903704 | 2.59E-22 | 4.60E-21 |
| C15orf48 | 1.831095 | 4.95124 | 2.34E-123 | 8.97E-121 |
| C1orf158 | 1.232658 | 4.822214 | 7.34E-46 | 4.36E-44 |
| C1orf168 | -1.39806 | 5.019499 | 9.44E-42 | 4.62E-40 |
| C1orf65 | -1.0414 | 4.618061 | 2.61E-16 | 3.13E-15 |
| C20orf195 | 1.501985 | 4.613188 | 2.09E-52 | 1.66E-50 |
| C21orf125 | -1.63764 | 5.129762 | 3.56E-60 | 3.49E-58 |
| C2orf66 | 1.060258 | 4.919411 | 1.37E-38 | 5.82E-37 |
| C4orf50 | -1.27177 | 4.852896 | 5.48E-30 | 1.48E-28 |
| C5orf38 | -1.10326 | 5.594116 | 1.69E-50 | 1.23E-48 |
| C6orf141 | 1.204449 | 5.162849 | 1.31E-64 | 1.43E-62 |
| C6orf15 | 2.243021 | 4.619924 | 3.38E-119 | 1.21E-116 |
| C6orf221 | -1.12218 | 4.572285 | 4.20E-17 | 5.31E-16 |
| C9orf122 | -1.20007 | 5.520479 | 2.23E-55 | 1.92E-53 |
| C9orf128 | -1.17298 | 4.568214 | 2.19E-18 | 3.03E-17 |
| C9orf44 | 1.553411 | 4.730479 | 8.20E-66 | 9.41E-64 |
| CA9 | 1.498499 | 5.42361 | 6.32E-120 | 2.32E-117 |
| CACNG2 | -1.54021 | 5.675321 | 1.30E-91 | 2.96E-89 |
| CAPN13 | -1.27086 | 4.749949 | 3.69E-26 | 8.20E-25 |
| CASP5 | 1.199077 | 4.675854 | 4.35E-36 | 1.60E-34 |
| CATSPER1 | 1.669678 | 4.515641 | 5.19E-56 | 4.56E-54 |
| CBLN1 | -1.17001 | 5.8459 | 2.64E-71 | 3.62E-69 |
| CCBE1 | -1.16397 | 4.786887 | 5.25E-24 | 1.03E-22 |
| CCDC19 | 1.223413 | 4.945607 | 2.27E-52 | 1.79E-50 |
| CCL18 | 1.22247 | 4.80829 | 2.44E-44 | 1.34E-42 |
| CCL20 | 1.752018 | 4.774014 | 2.62E-89 | 5.82E-87 |
| CCL26 | 1.267824 | 4.557805 | 5.24E-34 | 1.73E-32 |
| CD3D | 1.138009 | 4.909199 | 1.86E-43 | 9.76E-42 |
| CD70 | 2.255739 | 4.576992 | 1.24E-112 | 4.01E-110 |
| CD80 | 1.218232 | 4.740103 | 8.62E-41 | 3.98E-39 |
| CDH12 | -1.03284 | 5.324578 | 6.15E-36 | 2.24E-34 |
| CDH7 | -1.08392 | 5.190457 | 1.69E-33 | 5.43E-32 |
| CDH9 | -1.06658 | 5.336376 | 7.79E-38 | 3.18E-36 |
| CDHR5 | -1.52626 | 4.775299 | 6.44E-36 | 2.34E-34 |
| CEP170L | -1.98481 | 4.998718 | 9.58E-68 | 1.17E-65 |
| CES7 | -1.17608 | 4.59103 | 2.55E-19 | 3.74E-18 |
| CHP2 | -1.38627 | 4.575184 | 6.43E-24 | 1.26E-22 |
| CHRDL2 | 1.091361 | 5.025173 | 2.77E-45 | 1.59E-43 |
| CHRM2 | -1.13702 | 4.76829 | 7.50E-23 | 1.38E-21 |
| CHRNA9 | 1.718368 | 5.130829 | 2.27E-130 | 9.58E-128 |
| CIDEA | -1.69499 | 4.663179 | 4.09E-36 | 1.50E-34 |
| CKM | 1.208367 | 4.53553 | 3.10E-30 | 8.49E-29 |
| CLEC12A | 1.273053 | 4.997684 | 1.66E-60 | 1.64E-58 |
| CLEC5A | 1.111319 | 5.638911 | 3.06E-79 | 5.55E-77 |
| CPA4 | 1.248383 | 5.072809 | 3.87E-64 | 4.15E-62 |
| CSF3 | 1.273692 | 4.696785 | 7.15E-42 | 3.55E-40 |
| CSMD3 | -1.06789 | 6.205336 | 2.44E-83 | 4.85E-81 |
| CT45A1 | -1.49241 | 4.949878 | 3.27E-42 | 1.65E-40 |
| CTCFL | -1.13391 | 5.291037 | 1.78E-40 | 8.13E-39 |
| CXCL10 | 1.024448 | 5.6514 | 6.84E-68 | 8.55E-66 |
| CXCL6 | 1.423287 | 4.686041 | 1.07E-51 | 8.08E-50 |
| CXCR3 | 1.032067 | 4.755102 | 2.48E-29 | 6.49E-28 |
| CYP17A1 | -1.00513 | 4.512194 | 1.88E-13 | 1.79E-12 |
| CYP1A1 | -1.40012 | 4.948704 | 3.58E-39 | 1.54E-37 |
| CYP2A6 | -2.47486 | 4.711403 | 2.03E-64 | 2.19E-62 |
| CYP4B1 | -1.01974 | 4.901775 | 2.68E-22 | 4.75E-21 |
| DCAF12L2 | -1.74205 | 5.040485 | 4.50E-59 | 4.29E-57 |
| DEFA1B | 1.575528 | 4.587837 | 2.25E-55 | 1.94E-53 |
| DES | 1.112121 | 5.490272 | 3.12E-69 | 4.02E-67 |
| DGKK | -2.98045 | 5.09902 | 1.56E-122 | 5.85E-120 |
| DKFZP434H168 | -1.11545 | 4.785409 | 1.48E-22 | 2.68E-21 |
| DKFZp434J0226 | -1.81699 | 5.276113 | 1.02E-79 | 1.87E-77 |
| DKKL1 | 1.075112 | 4.645629 | 1.12E-27 | 2.73E-26 |
| DLK1 | 1.119293 | 4.820661 | 9.25E-38 | 3.74E-36 |
| DMRTA2 | 1.099855 | 5.540306 | 5.48E-71 | 7.39E-69 |
| DOC2B | -1.04823 | 5.373711 | 9.36E-39 | 4.00E-37 |
| DPEP1 | 1.199024 | 5.421973 | 9.94E-76 | 1.54E-73 |
| DRD5 | -1.23231 | 4.905377 | 2.52E-30 | 6.94E-29 |
| DSG2 | 1.130348 | 5.185219 | 8.18E-58 | 7.58E-56 |
| EBF2 | 1.396261 | 4.539298 | 2.40E-40 | 1.09E-38 |
| EEF1DP3 | -1.77947 | 4.543585 | 8.57E-33 | 2.64E-31 |
| EIF4E1B | -1.65903 | 4.927036 | 2.61E-48 | 1.71E-46 |
| ELANE | 1.213249 | 4.532244 | 5.06E-30 | 1.38E-28 |
| EN1 | 1.443115 | 5.303836 | 9.76E-99 | 2.57E-96 |
| EPHA6 | -1.42273 | 5.063132 | 5.46E-45 | 3.11E-43 |
| EPO | 1.059829 | 4.55696 | 7.64E-24 | 1.49E-22 |
| EREG | 1.485254 | 4.521135 | 1.76E-44 | 9.75E-43 |
| ESM1 | 1.329453 | 5.457585 | 9.27E-97 | 2.41E-94 |
| EXOC3L2 | 1.463739 | 5.064834 | 1.24E-88 | 2.72E-86 |
| F7 | -1.19754 | 5.241831 | 4.65E-42 | 2.33E-40 |
| FADS6 | -1.31677 | 4.904936 | 1.34E-33 | 4.34E-32 |
| FAM183A | 1.742815 | 4.672045 | 3.67E-77 | 6.07E-75 |
| FAM190A | -1.2752 | 5.220147 | 2.46E-45 | 1.42E-43 |
| FAM19A3 | 1.171408 | 4.954404 | 1.74E-48 | 1.15E-46 |
| FAM86B2 | 1.259369 | 4.974829 | 1.39E-57 | 1.28E-55 |
| FBXO39 | 1.724383 | 4.767098 | 9.28E-86 | 1.96E-83 |
| FBXO40 | -1.13043 | 4.833549 | 2.54E-24 | 5.08E-23 |
| FCGR2B | 1.008567 | 5.611989 | 3.22E-65 | 3.59E-63 |
| FCN3 | 1.313756 | 4.867941 | 3.78E-56 | 3.34E-54 |
| FER1L4 | 1.009074 | 5.173631 | 4.53E-46 | 2.72E-44 |
| FEZF1 | 1.498588 | 4.547292 | 3.09E-47 | 1.93E-45 |
| FKBP1AP1 | -1.19122 | 5.096349 | 1.05E-35 | 3.77E-34 |
| FLG | -1.07003 | 5.511694 | 1.26E-45 | 7.38E-44 |
| FMO1 | 1.000293 | 4.587714 | 3.45E-22 | 6.09E-21 |
| FOXE1 | -1.43762 | 4.569461 | 8.90E-25 | 1.84E-23 |
| FPR2 | 1.145853 | 5.106555 | 7.18E-55 | 6.15E-53 |
| FRMPD2 | -1.1108 | 5.308603 | 8.50E-40 | 3.72E-38 |
| FRRS1 | 1.006306 | 5.104445 | 1.09E-42 | 5.57E-41 |
| FSTL5 | -1.02129 | 5.95128 | 1.66E-62 | 1.74E-60 |
| FTHL3 | -1.78022 | 6.049236 | 1.01E-160 | 7.07E-158 |
| G0S2 | 1.092865 | 5.495441 | 2.99E-68 | 3.79E-66 |
| G6PC2 | -1.71765 | 4.901096 | 1.32E-49 | 9.13E-48 |
| GABARAPL3 | -1.42154 | 4.782003 | 8.43E-33 | 2.60E-31 |
| GAL | 1.125759 | 5.017928 | 6.72E-48 | 4.29E-46 |
| GALNT5 | 2.064257 | 4.798566 | 2.13E-128 | 8.37E-126 |
| GALNTL6 | -1.33456 | 4.970259 | 1.25E-36 | 4.73E-35 |
| GALR3 | -1.35201 | 4.558224 | 1.11E-22 | 2.02E-21 |
| GATA4 | 1.93016 | 4.655978 | 1.08E-92 | 2.49E-90 |
| GATA5 | -1.93048 | 4.631968 | 1.19E-41 | 5.76E-40 |
| GBP7 | -1.31684 | 4.711222 | 2.49E-26 | 5.58E-25 |
| GCSH | -1.21505 | 5.828952 | 2.07E-74 | 3.12E-72 |
| GDF15 | 1.019461 | 5.627676 | 1.15E-67 | 1.39E-65 |
| GDNF | -1.35996 | 4.831626 | 1.26E-32 | 3.85E-31 |
| GFRA3 | 1.019836 | 4.626422 | 3.59E-24 | 7.11E-23 |
| GGT8P | 1.345567 | 4.648697 | 4.17E-44 | 2.25E-42 |
| GJD2 | -1.25013 | 4.900603 | 1.18E-30 | 3.28E-29 |
| GLP1R | -1.69925 | 5.092411 | 5.23E-60 | 5.10E-58 |
| GLP2R | -1.52415 | 4.671779 | 1.53E-31 | 4.44E-30 |
| GLRA3 | -1.5516 | 5.460569 | 3.24E-76 | 5.21E-74 |
| GOLGA6B | -1.24995 | 4.912112 | 2.14E-31 | 6.17E-30 |
| GOLGA9P | -1.07063 | 4.517292 | 6.07E-15 | 6.43E-14 |
| GPA33 | 1.012717 | 4.610413 | 1.70E-23 | 3.24E-22 |
| GPR1 | 1.794774 | 4.854469 | 6.73E-104 | 1.86E-101 |
| GPR109A | 1.088862 | 4.685024 | 6.43E-30 | 1.73E-28 |
| GPR141 | 1.415422 | 4.607262 | 7.58E-46 | 4.49E-44 |
| GPR157 | 1.289196 | 4.520613 | 1.60E-33 | 5.14E-32 |
| GPR6 | -1.43205 | 4.798973 | 1.48E-33 | 4.77E-32 |
| GREB1L | -1.29723 | 5.578918 | 7.19E-66 | 8.37E-64 |
| GRIN2B | -1.09815 | 5.202408 | 1.27E-34 | 4.33E-33 |
| GSX2 | 1.501918 | 4.901519 | 4.40E-76 | 7.00E-74 |
| HAND2 | 1.102442 | 5.138705 | 2.51E-52 | 1.97E-50 |
| HAR1B | -1.55794 | 5.03428 | 3.38E-50 | 2.41E-48 |
| HBQ1 | -1.17337 | 4.997709 | 1.94E-31 | 5.62E-30 |
| HDC | 1.305146 | 4.541258 | 2.87E-35 | 1.01E-33 |
| HES2 | 1.288843 | 4.564059 | 2.27E-35 | 8.02E-34 |
| HGD | 1.584953 | 4.606691 | 6.20E-58 | 5.81E-56 |
| HILS1 | 1.133531 | 4.970884 | 4.15E-46 | 2.50E-44 |
| HIST1H2AM | 1.369397 | 4.508921 | 3.35E-37 | 1.31E-35 |
| HIST1H2BO | 1.183005 | 4.520619 | 3.92E-28 | 9.73E-27 |
| HIST1H3H | 1.211718 | 4.78213 | 8.12E-43 | 4.19E-41 |
| HMGA2 | 2.055328 | 4.815853 | 2.90E-130 | 1.19E-127 |
| HMX1 | -1.00338 | 5.891241 | 8.16E-58 | 7.58E-56 |
| HOTAIR | 2.655851 | 4.679986 | 1.45E-178 | 1.74E-175 |
| HOXA1 | 1.449009 | 5.063193 | 2.56E-85 | 5.32E-83 |
| HOXA10 | 1.391133 | 5.395834 | 2.56E-100 | 6.95E-98 |
| HOXA11 | 1.175233 | 5.015558 | 1.93E-52 | 1.55E-50 |
| HOXA2 | 2.344209 | 4.858344 | 2.79E-178 | 3.14E-175 |
| HOXA3 | 2.243496 | 5.015096 | 3.35E-197 | 8.08E-194 |
| HOXA4 | 2.032303 | 5.08686 | 4.82E-175 | 4.78E-172 |
| HOXA5 | 2.187553 | 5.037541 | 4.55E-192 | 8.53E-189 |
| HOXA6 | 2.155973 | 4.607486 | 9.10E-108 | 2.69E-105 |
| HOXA7 | 1.670983 | 5.328859 | 2.13E-137 | 9.97E-135 |
| HOXA9 | 1.92233 | 4.7783 | 2.53E-108 | 7.62E-106 |
| HOXB13 | 2.071527 | 4.709862 | 2.10E-115 | 7.22E-113 |
| HOXB2 | 1.185668 | 5.393244 | 5.77E-72 | 8.05E-70 |
| HOXB3 | 1.504618 | 5.365776 | 1.52E-114 | 5.14E-112 |
| HOXB4 | 1.597413 | 5.027343 | 2.70E-100 | 7.24E-98 |
| HOXB5 | 1.963495 | 4.515801 | 8.45E-78 | 1.43E-75 |
| HOXB6 | 1.2526 | 4.763815 | 4.27E-44 | 2.30E-42 |
| HOXC10 | 2.331909 | 5.041924 | 6.43E-219 | 2.17E-215 |
| HOXC11 | 2.677024 | 4.648114 | 3.94E-173 | 3.69E-170 |
| HOXC13 | 2.680112 | 4.664564 | 4.64E-178 | 4.90E-175 |
| HOXC5 | 1.688263 | 4.753573 | 1.21E-80 | 2.30E-78 |
| HOXC6 | 2.117289 | 4.919579 | 4.15E-157 | 2.41E-154 |
| HOXC8 | 2.286945 | 4.721413 | 5.63E-142 | 2.97E-139 |
| HOXC9 | 2.314256 | 4.779233 | 3.33E-157 | 2.08E-154 |
| HOXD10 | 1.993883 | 5.073152 | 2.77E-166 | 2.22E-163 |
| HOXD11 | 2.543747 | 4.897499 | 1.56E-218 | 4.38E-215 |
| HOXD13 | 2.384101 | 4.91041 | 2.34E-196 | 4.94E-193 |
| HOXD9 | 1.296937 | 5.265935 | 8.46E-84 | 1.72E-81 |
| HPD | 1.277999 | 5.130079 | 1.86E-70 | 2.49E-68 |
| HPSE2 | -1.79368 | 5.68908 | 1.02E-115 | 3.57E-113 |
| HRNR | -1.32566 | 5.018933 | 3.65E-38 | 1.52E-36 |
| HS3ST3B1 | 1.219469 | 4.987696 | 1.05E-54 | 8.89E-53 |
| HTR1A | -2.25592 | 5.070097 | 2.49E-86 | 5.31E-84 |
| HTR1E | -1.08628 | 4.840198 | 3.90E-23 | 7.32E-22 |
| HTR2C | -1.28322 | 5.382275 | 8.36E-53 | 6.78E-51 |
| IBSP | 2.254705 | 5.148545 | 4.36E-230 | 2.45E-226 |
| ID2B | -1.0292 | 5.230014 | 1.22E-32 | 3.72E-31 |
| IDO1 | 1.478608 | 4.76423 | 1.92E-62 | 2.00E-60 |
| IL13RA2 | 1.068528 | 5.679877 | 9.23E-76 | 1.44E-73 |
| IL1R2 | 1.231786 | 5.075517 | 1.71E-61 | 1.73E-59 |
| IL1RAPL1 | -1.01462 | 5.493614 | 3.12E-41 | 1.50E-39 |
| IL2RA | 1.859932 | 4.858598 | 3.86E-112 | 1.23E-109 |
| IMP5 | -1.35998 | 4.59035 | 1.14E-23 | 2.20E-22 |
| IRS4 | -1.09686 | 4.585478 | 4.41E-17 | 5.56E-16 |
| IRX2 | -1.27708 | 5.722768 | 1.32E-71 | 1.82E-69 |
| ISL2 | 1.439752 | 4.995862 | 7.03E-78 | 1.20E-75 |
| KCNB2 | -1.10398 | 5.169251 | 6.56E-34 | 2.16E-32 |
| KCNH6 | -1.02553 | 4.761279 | 2.92E-19 | 4.28E-18 |
| KCNJ15 | 1.361254 | 4.774277 | 1.13E-53 | 9.36E-52 |
| KHDRBS2 | -1.23204 | 5.475495 | 2.11E-55 | 1.84E-53 |
| KIAA1486 | -1.52055 | 5.155321 | 7.63E-55 | 6.50E-53 |
| KIF12 | -1.69391 | 4.561021 | 1.56E-31 | 4.53E-30 |
| KISS1R | 1.78491 | 4.694789 | 2.50E-83 | 4.90E-81 |
| KLHDC7A | 1.343362 | 4.612691 | 1.06E-41 | 5.14E-40 |
| KLK5 | -1.3341 | 4.567139 | 3.95E-22 | 6.93E-21 |
| KLRC2 | -1.08379 | 6.102376 | 1.42E-75 | 2.17E-73 |
| KLRC4 | -1.19518 | 5.479163 | 8.66E-52 | 6.64E-50 |
| KRT19 | -1.00912 | 5.03783 | 1.86E-25 | 4.02E-24 |
| KRT7 | 1.180326 | 4.953896 | 2.40E-49 | 1.62E-47 |
| KRT75 | 2.51221 | 4.572622 | 1.08E-137 | 5.20E-135 |
| KRT80 | 1.425406 | 4.693326 | 2.34E-52 | 1.84E-50 |
| KRT83 | -1.2841 | 4.578559 | 2.03E-21 | 3.36E-20 |
| LCE1E | -1.90167 | 4.51389 | 1.97E-34 | 6.68E-33 |
| LEMD1 | 1.101823 | 4.780006 | 1.43E-34 | 4.89E-33 |
| LGALS12 | 1.019969 | 4.557452 | 4.45E-22 | 7.76E-21 |
| LGALS9C | -1.12767 | 5.220574 | 2.90E-37 | 1.14E-35 |
| LHFPL5 | -1.01221 | 4.693334 | 2.18E-17 | 2.82E-16 |
| LHX5 | -1.64763 | 5.185205 | 3.01E-63 | 3.19E-61 |
| LILRA5 | 1.387064 | 4.905925 | 5.91E-65 | 6.47E-63 |
| LMX1A | -2.63198 | 4.673156 | 9.66E-66 | 1.10E-63 |
| LRIT2 | -1.43186 | 5.418471 | 4.55E-66 | 5.33E-64 |
| LRRC15 | 1.787748 | 4.727543 | 1.55E-87 | 3.36E-85 |
| LRRC8E | 1.108916 | 4.911319 | 8.71E-42 | 4.31E-40 |
| LRRN4 | -1.07069 | 4.826601 | 2.27E-22 | 4.06E-21 |
| LTF | 1.181107 | 5.915736 | 3.99E-114 | 1.32E-111 |
| MAB21L2 | 1.540808 | 4.581015 | 1.77E-52 | 1.42E-50 |
| MACC1 | 1.056466 | 5.124872 | 4.33E-48 | 2.82E-46 |
| MAP1LC3C | 1.536504 | 5.061724 | 5.23E-96 | 1.34E-93 |
| MATN4 | 1.238796 | 4.668349 | 5.92E-38 | 2.44E-36 |
| MCHR2 | -1.24214 | 4.828224 | 5.02E-28 | 1.24E-26 |
| MEOX2 | 1.470264 | 5.560537 | 1.99E-131 | 8.62E-129 |
| MGC12916 | 1.381302 | 4.609827 | 1.15E-43 | 6.11E-42 |
| MGC14436 | -1.04141 | 4.732672 | 5.04E-19 | 7.25E-18 |
| MIPOL1 | -1.01448 | 5.694615 | 2.42E-49 | 1.63E-47 |
| MIR155HG | 1.162084 | 5.178173 | 4.79E-62 | 4.92E-60 |
| MMP1 | 1.93509 | 4.663642 | 4.81E-94 | 1.14E-91 |
| MMP7 | 1.33156 | 5.152894 | 9.83E-79 | 1.71E-76 |
| MMP9 | 1.47409 | 5.608864 | 4.86E-138 | 2.48E-135 |
| MPZL3 | 1.073231 | 4.566938 | 6.56E-25 | 1.37E-23 |
| MSX2P1 | -2.09498 | 4.63472 | 1.94E-46 | 1.18E-44 |
| MYBPH | 1.309867 | 5.140228 | 6.55E-75 | 9.95E-73 |
| MYH6 | -1.60721 | 5.428177 | 6.14E-79 | 1.10E-76 |
| MYOD1 | -2.40703 | 4.822871 | 3.26E-70 | 4.30E-68 |
| NACAP1 | -1.75393 | 5.866252 | 3.62E-134 | 1.61E-131 |
| NBLA00301 | 1.123252 | 4.624883 | 2.03E-29 | 5.33E-28 |
| NCRNA00093 | -1.52869 | 5.464374 | 4.82E-76 | 7.60E-74 |
| NCRNA00164 | -1.32558 | 4.698919 | 2.81E-26 | 6.25E-25 |
| NDST4 | -1.97413 | 5.147564 | 9.46E-79 | 1.66E-76 |
| NKX2.5 | 2.035044 | 5.063389 | 3.14E-171 | 2.79E-168 |
| NKX2.8 | -1.01262 | 4.625769 | 6.07E-16 | 7.04E-15 |
| NKX3.2 | 1.015576 | 5.084443 | 9.02E-42 | 4.45E-40 |
| NT5C1A | -1.30802 | 5.211298 | 7.74E-47 | 4.77E-45 |
| OR2H2 | -1.08953 | 5.00468 | 3.69E-28 | 9.19E-27 |
| OR4N2 | -1.1534 | 5.079329 | 4.96E-33 | 1.56E-31 |
| OR4N4 | -1.95049 | 4.559789 | 5.03E-38 | 2.08E-36 |
| OR51E1 | 2.061263 | 4.889884 | 9.30E-144 | 5.06E-141 |
| OSR2 | 1.331204 | 5.046447 | 2.72E-70 | 3.62E-68 |
| OTOS | 1.033472 | 5.084971 | 2.93E-43 | 1.52E-41 |
| OTP | 2.30613 | 4.935472 | 1.71E-189 | 2.40E-186 |
| PABPC1L2A | -1.07947 | 5.129166 | 2.40E-31 | 6.92E-30 |
| PAR4 | -2.63913 | 4.608121 | 5.43E-60 | 5.26E-58 |
| PAX1 | -1.57861 | 5.341155 | 1.59E-69 | 2.07E-67 |
| PAX3 | 2.349959 | 4.737744 | 3.21E-153 | 1.81E-150 |
| PCDH11Y | -1.05735 | 5.353578 | 6.80E-38 | 2.79E-36 |
| PGA3 | -1.03613 | 5.156454 | 5.84E-30 | 1.57E-28 |
| PGR | -1.04495 | 5.494309 | 3.05E-43 | 1.58E-41 |
| PHLDA2 | 1.28659 | 5.17339 | 2.77E-76 | 4.49E-74 |
| PI3 | 2.38393 | 4.886334 | 3.62E-190 | 5.55E-187 |
| PIK3C2G | -1.54742 | 4.775212 | 1.15E-36 | 4.39E-35 |
| PITX1 | 1.120714 | 5.32112 | 3.47E-65 | 3.85E-63 |
| PLA2G2A | 2.582618 | 5.097905 | 6.56E-283 | 1.11E-278 |
| PLEK2 | 1.487812 | 5.188968 | 3.81E-105 | 1.07E-102 |
| PNPLA5 | -1.08089 | 4.611473 | 3.46E-17 | 4.40E-16 |
| PON1 | -1.34687 | 5.241866 | 2.88E-50 | 2.06E-48 |
| POSTN | 1.59894 | 5.645371 | 4.02E-169 | 3.39E-166 |
| PPIAL4C | -1.92597 | 5.358308 | 2.38E-95 | 5.90E-93 |
| PRG2 | 1.777123 | 4.534504 | 7.25E-66 | 8.38E-64 |
| PRKG2 | -1.48406 | 5.410827 | 5.28E-68 | 6.64E-66 |
| PRLHR | -2.35258 | 5.629138 | 3.60E-157 | 2.17E-154 |
| PRND | 1.096008 | 4.691917 | 1.58E-30 | 4.37E-29 |
| PROK2 | 1.166617 | 4.677941 | 3.62E-34 | 1.20E-32 |
| PRRX2 | 1.160063 | 4.75683 | 2.03E-37 | 8.02E-36 |
| PRTN3 | 1.008186 | 4.534787 | 8.58E-21 | 1.37E-19 |
| PTGER1 | 1.010589 | 4.561299 | 7.14E-22 | 1.23E-20 |
| PTPN22 | 1.131193 | 5.140909 | 1.76E-56 | 1.56E-54 |
| RANBP17 | -1.26676 | 5.72063 | 2.29E-72 | 3.25E-70 |
| RASEF | 1.096981 | 4.759996 | 1.45E-33 | 4.66E-32 |
| RBP3 | -1.58853 | 5.285473 | 2.13E-67 | 2.56E-65 |
| RD3 | -2.13838 | 4.56981 | 8.89E-44 | 4.73E-42 |
| RDM1 | 1.558335 | 4.782328 | 4.40E-71 | 5.98E-69 |
| RFPL4B | -1.35609 | 4.628698 | 7.08E-25 | 1.48E-23 |
| RPL13AP3 | -4.16658 | 5.065469 | 2.19E-164 | 1.68E-161 |
| RXFP1 | -1.0552 | 5.166522 | 3.28E-31 | 9.37E-30 |
| S100A12 | 1.356127 | 4.633678 | 1.53E-43 | 8.06E-42 |
| SAA1 | 2.713068 | 4.939023 | 5.78E-259 | 4.88E-255 |
| SAA2 | 2.88648 | 4.72894 | 2.73E-222 | 1.15E-218 |
| SBF1P1 | -1.2174 | 4.629278 | 2.17E-21 | 3.59E-20 |
| SCNN1B | 1.721318 | 4.987319 | 1.18E-111 | 3.70E-109 |
| SCNN1G | 1.475238 | 4.768765 | 3.39E-62 | 3.51E-60 |
| SCT | -1.78881 | 4.544705 | 2.31E-33 | 7.35E-32 |
| SERPINA5 | 1.094077 | 5.533221 | 1.16E-69 | 1.52E-67 |
| SGCZ | -1.0632 | 4.849585 | 1.96E-22 | 3.52E-21 |
| SHOX2 | 1.764564 | 5.355153 | 4.99E-158 | 3.24E-155 |
| SIRPG | 1.179203 | 4.56509 | 8.32E-30 | 2.22E-28 |
| SIT1 | 1.173261 | 4.755243 | 3.63E-38 | 1.51E-36 |
| SIX6 | 1.134338 | 4.697195 | 4.65E-33 | 1.46E-31 |
| SKINTL | 1.151742 | 4.510521 | 3.92E-26 | 8.68E-25 |
| SLAMF1 | 1.380054 | 4.542221 | 1.08E-39 | 4.73E-38 |
| SLAMF9 | 2.406268 | 4.609758 | 3.22E-134 | 1.47E-131 |
| SLC10A6 | 1.016519 | 4.680198 | 6.47E-26 | 1.42E-24 |
| SLC14A2 | -1.41435 | 5.238213 | 2.79E-53 | 2.28E-51 |
| SLC1A6 | -1.15199 | 5.887526 | 4.18E-72 | 5.87E-70 |
| SLC22A6 | -1.71445 | 5.671115 | 3.21E-109 | 9.84E-107 |
| SLC25A21 | -1.81492 | 5.073591 | 3.63E-65 | 4.00E-63 |
| SLC35D3 | -1.14114 | 4.767071 | 1.02E-22 | 1.85E-21 |
| SLC4A9 | -1.02116 | 4.5292 | 3.87E-14 | 3.88E-13 |
| SLC6A10P | -2.75919 | 5.281142 | 1.02E-137 | 5.04E-135 |
| SLC9A2 | -1.16652 | 5.463477 | 5.21E-50 | 3.68E-48 |
| SLITRK6 | -1.18258 | 4.793596 | 9.67E-25 | 1.99E-23 |
| SMCP | -2.62059 | 4.848855 | 1.21E-80 | 2.30E-78 |
| SMCR5 | -1.45075 | 5.014072 | 2.77E-44 | 1.51E-42 |
| SNORD115.26 | -2.28716 | 5.030982 | 6.82E-84 | 1.40E-81 |
| SOHLH1 | -1.10377 | 5.215253 | 2.62E-35 | 9.22E-34 |
| SP7 | -1.19278 | 4.989339 | 5.58E-32 | 1.66E-30 |
| SPAG17 | 1.178046 | 4.702551 | 4.75E-36 | 1.74E-34 |
| SPAG4 | 1.255852 | 5.278874 | 6.08E-81 | 1.18E-78 |
| SPDYE7P | -1.29504 | 4.89984 | 7.45E-33 | 2.32E-31 |
| SPINK8 | 1.405101 | 5.010552 | 1.19E-76 | 1.95E-74 |
| SSTR1 | -1.14432 | 5.982197 | 2.67E-77 | 4.45E-75 |
| SSTR3 | -1.13103 | 4.90172 | 1.92E-26 | 4.30E-25 |
| SSTR5 | -1.89223 | 4.817446 | 1.79E-51 | 1.33E-49 |
| STAC | 1.161162 | 5.456926 | 2.75E-73 | 4.00E-71 |
| STC1 | 1.007303 | 5.635097 | 2.18E-66 | 2.57E-64 |
| STL | -1.03286 | 4.777975 | 5.39E-20 | 8.20E-19 |
| STON1.GTF2A1L | -1.25538 | 4.880337 | 6.06E-30 | 1.63E-28 |
| SYCE1 | -1.43822 | 5.439815 | 9.62E-67 | 1.15E-64 |
| SYT10 | -2.53631 | 4.529331 | 9.85E-52 | 7.49E-50 |
| TARP | -1.14599 | 5.061379 | 2.02E-32 | 6.11E-31 |
| TDO2 | 1.04416 | 5.305522 | 1.24E-56 | 1.11E-54 |
| TDRD5 | -1.04864 | 4.887714 | 4.30E-23 | 8.04E-22 |
| TERT | 1.102646 | 4.798371 | 1.88E-35 | 6.69E-34 |
| TFAP2B | 1.480355 | 4.656011 | 5.91E-54 | 4.94E-52 |
| TFPI2 | 1.010605 | 5.071372 | 8.15E-41 | 3.78E-39 |
| TGM5 | 1.050298 | 4.942502 | 4.27E-38 | 1.77E-36 |
| TLX1NB | -1.31132 | 4.983712 | 3.42E-36 | 1.26E-34 |
| TM7SF4 | 1.080889 | 4.639568 | 1.18E-27 | 2.86E-26 |
| TMEM105 | -1.21645 | 4.510939 | 5.90E-18 | 7.92E-17 |
| TMEM61 | 1.100782 | 4.728268 | 2.08E-32 | 6.28E-31 |
| TNFSF14 | 1.654303 | 4.560179 | 1.05E-58 | 1.00E-56 |
| TOP1P2 | -3.77185 | 4.746461 | 1.96E-105 | 5.60E-103 |
| TP53TG5 | -1.70747 | 4.770505 | 1.41E-41 | 6.81E-40 |
| TREM1 | 1.452011 | 5.371002 | 7.28E-107 | 2.12E-104 |
| TREML2 | 1.433622 | 4.549669 | 2.57E-43 | 1.35E-41 |
| TRPM8 | 1.383539 | 5.265731 | 1.11E-95 | 2.80E-93 |
| TUBB8 | -1.16886 | 4.822549 | 2.53E-25 | 5.43E-24 |
| UCN2 | 1.245739 | 4.719789 | 3.24E-41 | 1.55E-39 |
| ULBP3 | 1.152365 | 5.111782 | 7.69E-57 | 6.90E-55 |
| VGLL2 | 2.012576 | 4.609763 | 2.57E-94 | 6.20E-92 |
| WBP11P1 | -1.74792 | 5.101506 | 7.04E-64 | 7.52E-62 |
| WDR87 | -1.56149 | 4.717299 | 1.45E-34 | 4.95E-33 |
| WISP1 | 1.037069 | 5.484075 | 3.43E-61 | 3.46E-59 |
| XG | 1.058249 | 4.552748 | 2.21E-23 | 4.19E-22 |
| XKR7 | -1.49336 | 5.28361 | 1.35E-60 | 1.34E-58 |
| ZNF676 | -1.39495 | 5.14578 | 2.27E-47 | 1.43E-45 |
| ZNF727 | -1.18795 | 5.363472 | 8.10E-47 | 4.97E-45 |
| ZNF804B | -1.27638 | 4.577968 | 2.41E-21 | 3.95E-20 |
| ZNF98 | -1.18355 | 5.137681 | 1.93E-36 | 7.20E-35 |
| ZSCAN10 | -1.30353 | 4.532085 | 1.86E-20 | 2.90E-19 |
