# Supplementary material for: A Risk Classification System With Five-Gene for Survival Prediction of Glioblastoma Patients
Source: Front Neurol. 2019 Jul 16;10:745. doi: 10.3389/fneur.2019.00745 (PMC6646669; doi:10.3389/fneur.2019.00745)
Supplement: Supplementary file 2 [file Table_2.docx]

Supplementary Table2. Selecting prognosis related genes in TCGA GBMLGG (RNA-seq) and HG-UG133A platforms using univariate Cox proportional hazards regression analysis.

| LGGGBM RNA-seq | | | HG-UG133A | | |
| --- | --- | --- | --- | --- | --- |
| gene | HR | P value | gene | HR | P value |
| FAM86B2 | 1.234708 | 0.001364 | RANBP17 | 0.404711 | 1.33E-08 |
| GPR1 | 1.144682 | 0.002144 | CLEC5A | 1.200823 | 1.45E-07 |
| G0S2 | 1.168931 | 0.00258 | POSTN | 1.131676 | 2.30E-05 |
| HOXC6 | 1.148533 | 0.002608 | FCGR2B | 1.125103 | 7.67E-05 |
| FER1L4 | 1.152599 | 0.00277 | HMX1 | 0.515961 | 0.000158 |
| RANBP17 | 0.844599 | 0.002951 | MEOX2 | 1.165741 | 0.000426 |
| HOXC10 | 1.100775 | 0.003358 | GREB1L | 0.678587 | 0.000668 |
| DES | 1.14901 | 0.003555 | IL13RA2 | 1.071169 | 0.000956 |
| LRRC8E | 1.171865 | 0.003826 | LTF | 1.049842 | 0.001684 |
| ARL9 | 1.162338 | 0.004337 | ABCC3 | 1.110837 | 0.001817 |
| HOXB2 | 1.116386 | 0.005232 | IBSP | 1.128088 | 0.001872 |
| SPAG4 | 1.190669 | 0.00525 | NDST4 | 0.661554 | 0.001959 |
| HOXB5 | 1.114946 | 0.006232 | HOXC10 | 1.11233 | 0.003888 |
| KHDRBS2 | 0.864064 | 0.007478 | TREM1 | 1.082214 | 0.004862 |
| HOXB3 | 1.108106 | 0.007622 | HOXC13 | 1.278241 | 0.005127 |
| FBXO39 | 1.196379 | 0.008113 | STC1 | 1.115817 | 0.005136 |
| SLC25A21 | 0.820608 | 0.008304 | CBLN1 | 0.670779 | 0.006914 |
| SERPINA5 | 1.160908 | 0.009 | MYOD1 | 0.606449 | 0.007427 |
| HOXC13 | 1.093454 | 0.009095 | SLC25A21 | 0.571362 | 0.007878 |
| TNFSF14 | 1.160269 | 0.009495 | PRKG2 | 0.643997 | 0.013243 |
| TFAP2B | 1.096348 | 0.009992 | HOXC11 | 1.253573 | 0.015925 |
| ZNF98 | 0.847484 | 0.010662 | HOXD10 | 1.203975 | 0.015999 |
| STC1 | 1.181252 | 0.011492 | PLA2G2A | 1.051261 | 0.016002 |
| HOTAIR | 1.096352 | 0.011518 | CXCL10 | 1.060497 | 0.01722 |
| TMEM61 | 1.150358 | 0.01162 | STAC | 1.079287 | 0.021099 |
| HMGA2 | 1.11158 | 0.012089 | G0S2 | 1.096754 | 0.021245 |
| AQP5 | 1.111266 | 0.01288 | IL1R2 | 1.088182 | 0.02496 |
| HOXC8 | 1.098949 | 0.013617 | HOXD11 | 1.146598 | 0.025262 |
| KRT80 | 1.154481 | 0.014811 | CCL20 | 1.069486 | 0.025422 |
| HOXC9 | 1.099959 | 0.014835 | IL1RAPL1 | 0.821712 | 0.025865 |
| HOXB13 | 1.080819 | 0.015222 | HOXA5 | 1.06453 | 0.031949 |
| DSG2 | 1.082629 | 0.015389 | DES | 1.533564 | 0.036664 |
| ANKK1 | 1.154162 | 0.015622 | HOXB2 | 1.068185 | 0.037119 |
| MEOX2 | 1.101821 | 0.017013 | SSTR5 | 0.654706 | 0.037877 |
| GATA4 | 1.091223 | 0.017155 | KHDRBS2 | 0.811361 | 0.038058 |
| C20orf195 | 1.187958 | 0.018785 | FPR2 | 1.421367 | 0.03871 |
| HOXC11 | 1.087201 | 0.020287 | WISP1 | 1.184854 | 0.041928 |
| KISS1R | 1.108332 | 0.020645 | PI3 | 1.052427 | 0.04317 |
| RBP3 | 0.862927 | 0.021632 | DPEP1 | 0.847258 | 0.045941 |
| HOXD11 | 1.105042 | 0.023332 |  |  |  |
| AGTR1 | 1.13646 | 0.02347 |  |  |  |
| KRT7 | 1.12044 | 0.025434 |  |  |  |
| ADAMTS20 | 0.836021 | 0.025714 |  |  |  |
| LRRC15 | 1.102095 | 0.026168 |  |  |  |
| GPR157 | 1.180409 | 0.029377 |  |  |  |
| MIR155HG | 1.167365 | 0.030972 |  |  |  |
| RDM1 | 1.150792 | 0.031192 |  |  |  |
| FCGR2B | 1.112122 | 0.031821 |  |  |  |
| HOXB4 | 1.076241 | 0.037203 |  |  |  |
| ULBP3 | 1.165995 | 0.038087 |  |  |  |
| CA9 | 1.079968 | 0.040162 |  |  |  |
| CPA4 | 1.122046 | 0.040404 |  |  |  |
| SAA2 | 1.05654 | 0.041179 |  |  |  |
| POSTN | 1.064763 | 0.042639 |  |  |  |
| CLEC5A | 1.133886 | 0.044475 |  |  |  |
| ABCC3 | 1.103241 | 0.044817 |  |  |  |
| HOXD10 | 1.088086 | 0.049313 |  |  |  |
| TREML2 | 1.152127 | 0.049815 |  |  |  |
