# Supplementary material for: A Risk Classification System With Five-Gene for Survival Prediction of Glioblastoma Patients
Source: Front Neurol. 2019 Jul 16;10:745. doi: 10.3389/fneur.2019.00745 (PMC6646669; doi:10.3389/fneur.2019.00745)
Supplement: Supplementary file 3 [file Table_3.docx]

Supplementary Table3. DEGs between the high- risk group and low- risk group.

| Gene | logFC | logCPM | P Value | FDR |
| --- | --- | --- | --- | --- |
| PTX3 | 0.349219 | 7.363427 | 1.53E-14 | 1.19E-10 |
| CLEC5A | 0.346031 | 7.169063 | 2.64E-12 | 6.82E-09 |
| POSTN | 0.333158 | 7.135331 | 3.53E-11 | 5.46E-08 |
| LTF | 0.31604 | 7.498575 | 1.84E-13 | 7.13E-10 |
| PLA2G2A | 0.31357 | 7.2521 | 5.03E-11 | 6.48E-08 |
| LOX | 0.290779 | 7.253296 | 1.07E-09 | 1.04E-06 |
| F13A1 | 0.274637 | 7.331048 | 2.64E-09 | 2.27E-06 |
| PI3 | 0.26479 | 6.978373 | 9.21E-07 | 0.000356 |
| FCGR2B | 0.261974 | 7.20312 | 7.93E-08 | 4.09E-05 |
| NNMT | 0.254465 | 7.589184 | 7.61E-10 | 8.41E-07 |
| TREM1 | 0.252701 | 7.254701 | 1.17E-07 | 5.64E-05 |
| CHI3L1 | 0.251567 | 7.83314 | 1.80E-11 | 3.48E-08 |
| IL8 | 0.248677 | 7.328685 | 7.47E-08 | 4.09E-05 |
| CXCL14 | 0.241107 | 7.523066 | 1.45E-08 | 1.02E-05 |
| PDPN | 0.241071 | 7.452887 | 3.62E-08 | 2.15E-05 |
| CHI3L2 | 0.240972 | 7.472819 | 2.71E-08 | 1.75E-05 |
| PLA2G5 | 0.238262 | 7.235819 | 7.07E-07 | 0.000304 |
| CCL2 | 0.233231 | 7.604382 | 1.42E-08 | 1.02E-05 |
| SERPINE1 | 0.224916 | 7.349054 | 8.74E-07 | 0.000356 |
| TDO2 | 0.224174 | 7.04445 | 1.91E-05 | 0.00462 |
| PLAU | 0.221687 | 7.085193 | 1.65E-05 | 0.004255 |
| COL5A1 | 0.220181 | 7.193874 | 6.95E-06 | 0.00215 |
| COL6A3 | 0.218909 | 7.323578 | 2.34E-06 | 0.000788 |
| ABCC3 | 0.218461 | 7.159122 | 1.12E-05 | 0.003185 |
| C21orf62 | 0.217102 | 7.243444 | 5.68E-06 | 0.001832 |
| LIF | 0.213988 | 6.948275 | 9.20E-05 | 0.013681 |
| CD163 | 0.211315 | 7.603049 | 2.78E-07 | 0.000126 |
| DIRAS3 | 0.208636 | 7.260325 | 1.14E-05 | 0.003185 |
| CHRNA9 | 0.203396 | 7.048563 | 9.91E-05 | 0.014194 |
| C5AR1 | 0.202901 | 7.161331 | 4.39E-05 | 0.007723 |
| HP | 0.200734 | 7.118375 | 7.44E-05 | 0.011738 |
| CA12 | 0.199383 | 7.364335 | 1.15E-05 | 0.003185 |
| PLAUR | 0.198723 | 7.236539 | 3.60E-05 | 0.007142 |
| SLN | 0.193339 | 7.342845 | 2.46E-05 | 0.005599 |
| RNASE2 | 0.19298 | 7.299487 | 3.70E-05 | 0.007154 |
| FCGBP | 0.190774 | 7.68912 | 1.48E-06 | 0.000522 |
| HSPA6 | 0.189664 | 7.054775 | 0.000274 | 0.030426 |
| CHL1 | 0.186121 | 7.497996 | 1.51E-05 | 0.004015 |
| MRC1 | 0.185791 | 7.082283 | 0.000316 | 0.033071 |
| SLPI | 0.185468 | 7.320592 | 6.54E-05 | 0.010764 |
| HOXC10 | 0.185035 | 7.044762 | 0.000415 | 0.038377 |
| SYNPO | 0.185022 | 7.227105 | 0.000127 | 0.017185 |
| ARSJ | 0.184035 | 7.080949 | 0.000357 | 0.035907 |
| ANGPTL4 | 0.183864 | 7.325352 | 6.93E-05 | 0.01116 |
| PCSK1 | 0.183601 | 7.179663 | 0.000195 | 0.024691 |
| RCAN1 | 0.18195 | 7.330899 | 8.12E-05 | 0.012567 |
| TAGLN | 0.181595 | 7.537414 | 1.76E-05 | 0.004389 |
| FMOD | 0.180821 | 7.190744 | 0.000235 | 0.027971 |
| COL3A1 | 0.175969 | 7.5149 | 3.83E-05 | 0.007235 |
| ACSS3 | 0.175269 | 7.220555 | 0.000301 | 0.031917 |
| TNFAIP6 | 0.172092 | 7.391619 | 0.00013 | 0.017395 |
| ADM | 0.172055 | 7.636307 | 2.24E-05 | 0.005256 |
| STEAP3 | 0.168939 | 7.26784 | 0.000368 | 0.036522 |
| SCG2 | 0.168379 | 7.55935 | 5.70E-05 | 0.009588 |
| HAMP | 0.168283 | 7.314323 | 0.00029 | 0.031146 |
| NR2E1 | 0.167501 | 7.352132 | 0.000248 | 0.028186 |
| EMP3 | 0.167268 | 7.663217 | 3.05E-05 | 0.006214 |
| DPYD | 0.167258 | 7.52355 | 8.41E-05 | 0.012757 |
| CFI | 0.165981 | 7.398795 | 0.000216 | 0.02662 |
| S100A8 | 0.164569 | 7.26738 | 0.000527 | 0.045754 |
| EFEMP1 | 0.164241 | 7.67471 | 3.94E-05 | 0.007253 |
| SOD2 | 0.16398 | 7.423885 | 0.000217 | 0.02662 |
| MOXD1 | 0.162635 | 7.323769 | 0.000446 | 0.040132 |
| FBLN5 | 0.162357 | 7.297296 | 0.000538 | 0.045754 |
| CYR61 | 0.162309 | 7.482963 | 0.000178 | 0.023007 |
| TGFBI | 0.161814 | 7.70975 | 4.03E-05 | 0.007253 |
| SLC2A3 | 0.16143 | 7.301307 | 0.000558 | 0.045944 |
| FABP7 | 0.161365 | 7.765915 | 2.69E-05 | 0.005939 |
| HRH1 | 0.160653 | 7.312513 | 0.000553 | 0.045944 |
| GLIPR1 | 0.158838 | 7.355796 | 0.000514 | 0.045157 |
| SERPINA1 | 0.158404 | 7.399618 | 0.000417 | 0.038377 |
| COL5A2 | 0.154317 | 7.495068 | 0.000339 | 0.034526 |
| SERPING1 | 0.15251 | 7.502505 | 0.000384 | 0.036667 |
| TMEM176B | 0.15199 | 7.51776 | 0.000376 | 0.036667 |
| VEGFA | 0.15105 | 7.462072 | 0.000542 | 0.045754 |
| C1S | 0.150109 | 7.628536 | 0.000232 | 0.027971 |
| C1R | 0.150059 | 7.598443 | 0.000275 | 0.030426 |
| AEBP1 | 0.147858 | 7.59751 | 0.000334 | 0.034497 |
| GAP43 | 0.143217 | 7.623889 | 0.000435 | 0.039589 |
| ALOX5AP | 0.142057 | 7.674994 | 0.000384 | 0.036667 |
| IGFBP2 | 0.141885 | 7.7275 | 0.000285 | 0.031044 |
| CD14 | 0.139646 | 7.640919 | 0.000574 | 0.0467 |
| NAMPT | 0.135923 | 7.751348 | 0.000453 | 0.040306 |
| NET1 | -0.17304 | 7.142696 | 0.000544 | 0.045754 |
| SH3GL2 | -0.17715 | 7.151641 | 0.000398 | 0.037532 |
| AKR1C3 | -0.17859 | 7.269279 | 0.000161 | 0.021105 |
| AKR1C1 | -0.18361 | 7.14298 | 0.000241 | 0.028186 |
| KCNK1 | -0.18991 | 7.066555 | 0.000246 | 0.028186 |
| FERMT1 | -0.1961 | 7.13456 | 9.79E-05 | 0.014194 |
| INA | -0.19858 | 7.07179 | 0.000125 | 0.017185 |
| TOX3 | -0.20079 | 7.238626 | 2.91E-05 | 0.006214 |
| AKR1C2 | -0.2024 | 7.037004 | 0.000113 | 0.015873 |
| UGT8 | -0.20802 | 7.096548 | 4.84E-05 | 0.008327 |
| FGF13 | -0.20861 | 7.146915 | 3.03E-05 | 0.006214 |
| SNAP91 | -0.25369 | 7.060569 | 1.05E-06 | 0.000388 |
