# Supplementary material for: A Risk Classification System With Five-Gene for Survival Prediction of Glioblastoma Patients
Source: Front Neurol. 2019 Jul 16;10:745. doi: 10.3389/fneur.2019.00745 (PMC6646669; doi:10.3389/fneur.2019.00745)

A Risk Classification System with Five-gene for Survival Prediction of Glioblastoma Patients

Yulin Wang[1], Xin Liu[2], Gefei Guan[3], Weijiang Zhao[4*] and Minghua Zhuang[1]*

[1] Department of Neurosurgery, The First Affiliated Hospital of Shantou University Medical College, Shantou, Guangdong 515041, China

[2] Department of stomatology, The First Affiliated Hospital of Shantou University Medical College, Shantou, Guangdong 515041, China

[3] Department of Neurosurgery, The First Hospital of China Medical University, Shenyang, Liaoning 110001, China

[4] Center for Neuroscience, Shantou University Medical College, Shantou, Guangdong 515041, China

Supplementary figure 1. ROC curve and calibration curve were used to evaluate the efficiency of the nomogram in primary cohort

(A) ROC curve was used to evaluate the efficiency of the clinical predictive model in the training cohort. (B-D) The calibration curves for predicting patient survival at 1, 3 and 5 years in the training cohort.

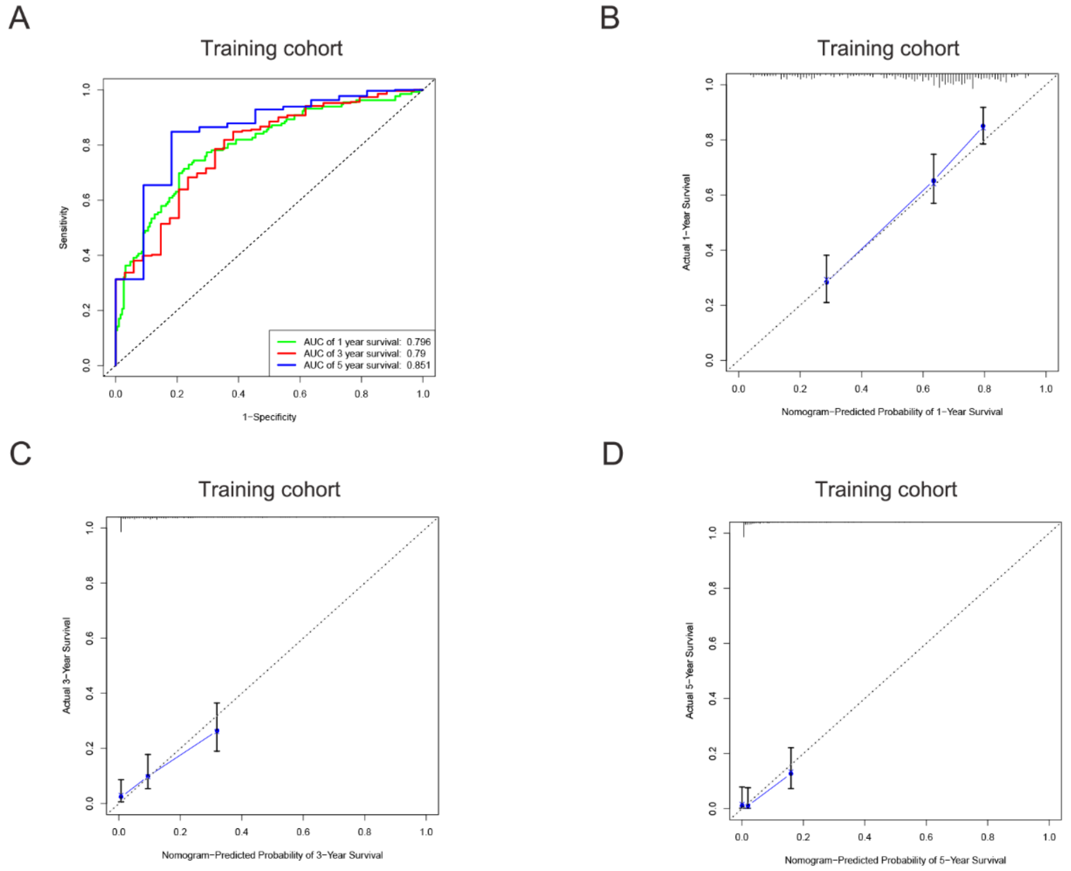

Supplement: Supplementary file 4 [file Image_1.pdf]
